# Production, Characterization and Biocompatibility Evaluation of Collagen Membranes Derived from Marine Sponge *Chondrosia reniformis* Nardo, 1847

**DOI:** 10.3390/md16040111

**Published:** 2018-03-29

**Authors:** Marina Pozzolini, Sonia Scarfì, Lorenzo Gallus, Maila Castellano, Silvia Vicini, Katia Cortese, Maria Cristina Gagliani, Marco Bertolino, Gabriele Costa, Marco Giovine

**Affiliations:** 1 Department of Earth, Environment and Life Sciences (DISTAV), University of Genova, Via Pastore 3, 16132 Genova, Italy; soniascarfi@unige.it (S.S.); galluslorenzo@gmail.com (L.G.); marco.bertolino@edu.unige.it (M.B.); gabriele.costa@edu.unige.it (G.C.); mgiovine@unige.it (M.G.); 2 Department of Chemistry and Industrial Chemistry (DCCI), University of Genova, Via Dodecaneso 31, 16146 Genova, Italy; maila@chimica.unige.it (M.C.); silvia.vicini@unige.it (S.V.); 3 Department of Experimental Medicine (DIMES), Human Anatomy Section, University of Genova, Via De Toni 14, 16132 Genova, Italy; cortesek@unige.it (K.C.); gagliani@unige.it (M.C.G.)

**Keywords:** collagen, Porifera, biomaterials, tissue engineering, membranes

## Abstract

Collagen is involved in the formation of complex fibrillar networks, providing the structural integrity of tissues. Its low immunogenicity and mechanical properties make this molecule a biomaterial that is extremely suitable for tissue engineering and regenerative medicine (TERM) strategies in human health issues. Here, for the first time, we performed a thorough screening of four different methods to obtain sponge collagenous fibrillar suspensions (FSs) from *C. reniformis* demosponge, which were then chemically, physically, and biologically characterized, in terms of protein, collagen, and glycosaminoglycans content, viscous properties, biocompatibility, and antioxidant activity. These four FSs were then tested for their capability to generate crosslinked or not thin sponge collagenous membranes (SCMs) that are suitable for TERM purposes. Two types of FSs, of the four tested, were able to generate SCMs, either from crosslinking or not, and showed good mechanical properties, enzymatic degradation resistance, water binding capacity, antioxidant activity, and biocompatibility on both fibroblast and keratinocyte cell cultures. Finally, our results demonstrate that it is possible to adapt the extraction procedure in order to alternatively improve the mechanical properties or the antioxidant performances of the derived biomaterial, depending on the application requirements, thanks to the versatility of *C. reniformis* extracellular matrix extracts.

## 1. Introduction

Collagen is the most abundant protein in animals; in particular, it is involved in the formation of a variety of fibrillar networks, providing the structural integrity of the tissues. This molecule, and its derived gelatin, is used as a biomaterial for tissue engineering and regenerative medicine (TERM) purposes, thanks to its low immunogenicity and mechanical properties. Collagen-derived biomaterials can be obtained in two ways: (i) by preserving as much as possible the shape of the original tissue via isolation of a de-cellularized extracellular matrix (ECM); or, (ii) by in vitro polymerization of soluble tropo-collagen, alone or combination with other ECM components, such as glycosaminoglycans (GAGs) [1,2], elastin [3,4] or chitosan [5,6]. These additions are meant to improve the enzymatic resistance and mechanical properties of the derived biomaterial. According to their application, collagen-based biomaterials can be used in the form of (i) collagen sponges, employed as three-dimensional (3-D) scaffolds in bone and cartilage repair; (ii) injectable hydrogels for drug delivery; and, (iii) two-dimensional (2-D) thin films or membranes suitable in wound dressing, dural closure, reinforcement of a compromised tissue and guided tissue regeneration [7]. The sources of collagen for these applications are mainly waste of bovine and porcine skin and bones. However, the risk of BSE (bovine spongiform encephalopathy), TSE (transmissible spongiform encephalopathy), and religious constraints (e.g., avoidance of porcine derivatives), has recently led to considering different sources of collagen. The most intriguing and promising ones come from the marine environment (for a review see [8]). In order to obtain economically sustainable amounts of these molecules, particular attention has been paid to collagens derived from fish or mollusks waste of food industry [9,10]. Besides, studies have also been done on collagens that were extracted from marine invertebrates, such as jellyfishes, generating real “blooms”, in particular, environmental or seasonal conditions [11,12]. 

Marine sponges (Porifera) are the oldest metazoan group still extant on our planet [13]. Unlike other animal groups, they are characterized by a simple level of organization, they lack real tissues and organs, and are only formed by specialized cells types (e.g., choanocytes and pinacocytes [14]). These cells are embedded in a complex 3-D matrix network that is rich in collagen, which, in the Demospongiae class, is generically referred to as spongin [15]. It is formed by proteoglycans, glycoproteins, conventional striated collagen fibrils, and thin collagen microfilaments forming large structures that may be associated to siliceous spicules, or, in horny sponges, to foreign materials [16]. Although still being poorly characterized at the molecular level [17,18], spongin extracts [19], or sponge intact skeletons [20,21,22] provide a suitable framework for mammalian cell attachment and for migration and the proliferation of osteoprogenitor cells. These features reveal a promising biomaterial for bone repair. Furthermore, new fields of application of spongin are also arising, such as its use as supports for the immobilization of dyes [23,24,25,26,27,28,29,30,31,32,33]. Interestingly, in the Hexatinellida, a new unexpected structural role of collagen has been recently described. Indeed, this protein was identified within the basal siliceous spicules of the glass sponge *Hyalonema sieboldii* Gray, 1835 [34,35], stimulating further research on sponge collagen for a better comprehension of the skeletal structures of these deep-sea organisms.

Sponges are also organisms with a high biotechnological potential, since they are a relevant marine source of bioactive compounds. These animals, in fact, have developed a large variety of secondary metabolites for their defense from competitors, predators, and pathogens. The types of molecules produced are mainly nucleosides, toxins, and terpenoids with antibacterial, antifungal, or antiviral activity [36]. Notably, of the 18,000 marine natural bioactive products that have described until now in the pharmaceutical field, more than 30% have been isolated from sponges [37]. Unfortunately, wild sponge populations are frequently insufficient or inaccessible to produce sustainable commercial quantities of metabolites of interest. Thus, various mariculture [38,39] and in vitro cell culture [40,41] methods have been developed to fully exploit their interesting pharmaceutical features. In this perspective, the waste of sponge biomasses remaining after bioactive compound extraction could be used as a source of marine collagen.

One of the most studied sponge collagen is derived from the *Chondrosia reniformis* species, which is a member of the Demospongiae class. Its marine habitat includes a broad geographic (and bathymetric) range of marine environments, among which is the Mediterranean Sea. The body of *C. reniformis* is mainly made of tightly packed collagenous fibres that are lacking the typical needle-like silica spicules that strengthen the body of most other species of the same class. Its collagen is characterized by a unique dynamic plasticity due to the ability to reversibly alter its viscoelastic properties in an extraordinary short-time span. These features seem to be under the control of a calcium-dependent extracellular aggregating factor [42,43]. In the past, this collagen was partially biochemically characterized [44,45], and, more recently, some of its gene sequences were identified [46,47], as well as that of a collagen-maturation enzyme [48]. Moreover, an ultra-structural study was performed thanks to its partial solubilization [49]. Its biocompatibility on human skin has already been evaluated [50], and its use in the form of nanoparticles as carriers and coatings for drug preparations has been also described [51,52]. Notwithstanding, the use of *C. reniformis* collagen for membranous scaffold production in TERM applications has never been reported.

The aim of the present work was to evaluate the performance of various types of *C. reniformis* collagen extracts for the production of membranous scaffolds for TERM strategies. For this purpose, intact collagen fibrillar extracts were obtained, using four different methods that were previously described in literature [46,53,54,55]. Following their physico-chemical and biological characterization, the four extracts were used for the generation of collagenous membranes. Finally, the obtained membranes were characterized, and their biocompatibility evaluated, in order to establish the best extraction procedure to generate suitable tools for TERM applications.

## 2. Results

### 2.1. Fibrillar Collagen Suspensions Extraction and Characterization

**Scheme 1:**
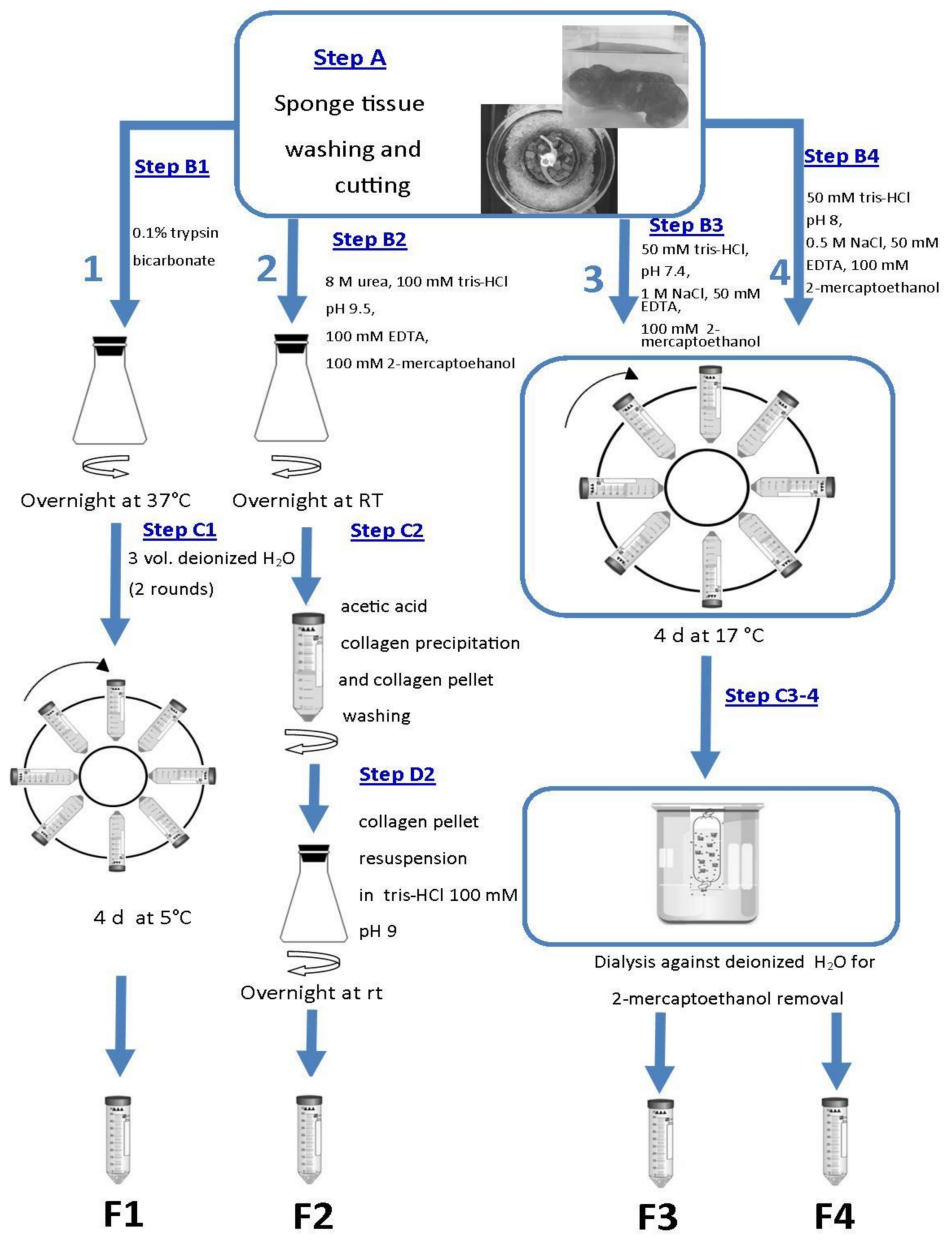
Extraction methods used. Schematic representation of the four extraction procedures used to obtain four different sponge fibrillar suspensions (F1–F4) as detailed in Section 4.1. Method 1 from [53]; method 2 from [54]; method 3 from [46]; and, method 4 from [55].

Fibrillar collagen suspensions (FS) were extracted from *C. reniformis* tissue using four different methods, as indicated in Scheme 1, in order to evaluate the more suitable for collagenous membrane production. The total soluble proteins, collagen and GAGs content were then quantified in each FS. Table 1 reports the total yields obtained in each extract (from F1 to F4) in terms of FS, collagen, and GAGs, expressed as total mg/g of dry sponge tissue. Conversely, Table 2 shows a comparison of the composition by the quantification of total soluble proteins, collagen, and GAGs, which were calculated as weight percentage with respect to the total FS content of each extract. Moreover, a collagen/GAGs ratio (R_C/GAG_) was calculated in order to establish the optimal collagen/sugar composition for the further membrane production.

**Figure 1:**
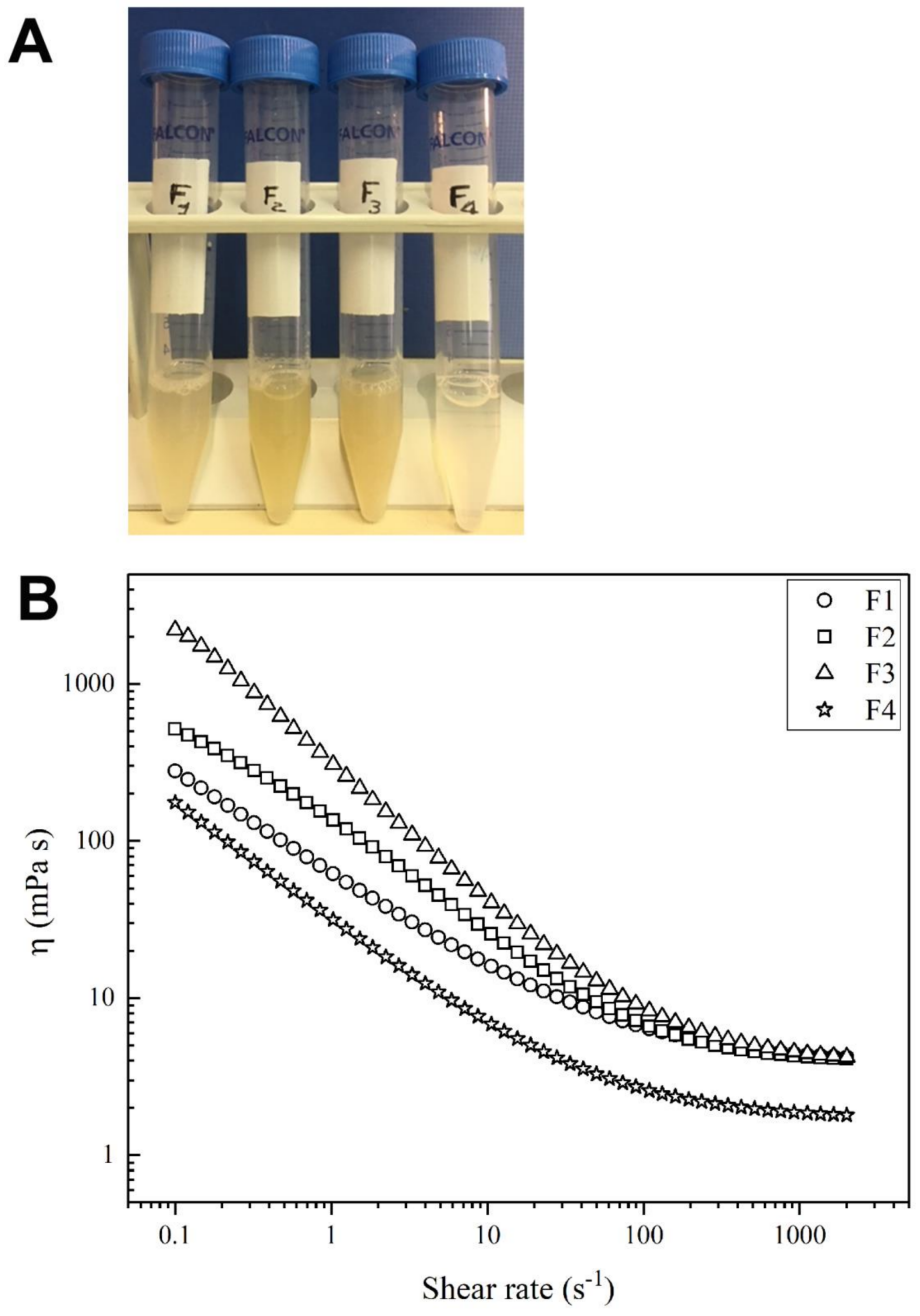
Fibrillar suspensions (FS) appearance and viscosity test. (**A**) Appearance of F1-F4 sponge FSs obtained through the four different extraction methods summarized in Scheme 1; (**B**) Flow sweep curves obtained in the viscosity tests of the four FSs by rheological measurements (for methods see Section 4.3.6). Curves were fitted by the Carreau-Gahleitner model (Mezger, 2006) as shown in Equation: $\frac{\eta -\eta_{\infty }}{\eta_{0}-\eta_{\infty }}=\;\frac{1}{{\left(1+{\left(a\cdot \;\dot{\gamma }\right)}^{b}\right)}^{P}}$ where η is the shear viscosity, η_∞_ is the infinity-shear viscosity, η_0_ is the zero-shear viscosity, a is the Carreau constant, b is the Gahleitner exponent, and P is the Carreau exponent. The experimental values of η_0_ and η_∞_ are shown in Table 3.

F1 extract was obtained, as described by Gross et al., 1956 [53], by an overnight trypsin digestion at 37 °C in order to remove non collagenous proteins as much as possible. The digested sponge tissue was then subjected to two rounds of water extraction at 5 °C. After the second step of water extraction, the resulted sponge tissue completely dissolved. The collagen extract resulted viscous and light colored (Figure 1A). The yield of F1 suspension had the lowest values with respect to the yields of the other FS extracts (Table 1, column 3). The yield in collagen had intermediate values, while the GAGs had the lowest values of extraction with respect to the values that were obtained in the other extracts (Table 1, columns 4 and 5, respectively). Conversely, in terms of percentages, F1 suspension resulted composed almost all of the soluble proteins, half of which were collagen (Table 2, columns 2 and 3, respectively). Finally, the R_C/GAG_ was of 14.95, the highest value observed with respect to the other FS extracts (Table 2).

F2 extract obtained, as described previously by Diehl-Seifert et al. (1985) [54], was characterized by sponge tissue extraction under alkaline and reducing conditions in 8 M urea. The concentrated urea was used to improve fibre dissolving, whereas 2-mercapto-ethanol was used to ensure the intra and interchain disulfide collagen bridge break. At the end of the 24 h incubation at room temperature (RT), the sponge tissue was not completely dissolved and the insoluble fraction was removed by centrifugation. The collagen fibres were then precipitated by lowering the pH with the addition of acetic acid. The repeated centrifugation steps during the washings were necessary to neutralize the pH, but made the suspension less homogeneous than the others (Figure 1A), with several aggregates that necessitated a further homogenization step. It is to note that, in these extraction conditions the collagen retained in the F2 suspension had the lowest yield respect to the other extracts (Table 1). Furthermore, less than half of the F2 suspension was composed of soluble proteins, of which only the 16% was collagen (Table 2). Conversely, the percentage of GAGs extracted was the highest with respect to the other FSs, and consequently, the R_C/GAG_ was the lowest (1.18), amounting to less than 1/10 of the F1 R_C/GAG_ (Table 2, columns 4 and 6, respectively).

F3 and F4 extracts were obtained with a similar procedure [46,55], in both cases in fact, sponge fragments were incubated in an orbital shaker disk for four days at 17 °C in the presence of 2-mercapto-ethanol and EDTA. The only differences were that in F3 the pH was 7.4, whereas in F4, it was adjusted to 8.0, and that for F3 extraction, 1 M NaCl was used, while for F4, the NaCl concentration was only of 0.5 M. With the F3 extraction method, complete sponge tissue dissolution was observed at the end of the 4 day incubation. Conversely, using the F4 extraction method some insoluble tissue fragments were still present. F3 final extract resulted viscous and lightly colored, quite similar to F1, while F4 resulted clearer than the other FSs (Figure 1A). With these two extraction methods, the total yields of the two suspensions were the highest with respect to the other two methods (Table 1, column 3). Moreover, the F3 method showed the highest collagen and GAGs yields with respect to all of the other extraction procedures (Table 1, columns 4 and 5, respectively). For what concerns protein percentages, with the F3 method, the suspension obtained was composed of more than 60% of soluble proteins, of which nearly the 50% was collagen, while in the F4 method, less than 30% of the suspension was composed of soluble proteins, of which the collagen amounted to 60% (Table 2, column 2 and 3, respectively). Finally, the R_C/GAG_ was higher in the F3 suspension, and was similar to the F1 value, and lower in the F4, pointedly nearly a half with respect to F1 and F3 (Table 2, column 6).

### 2.2. Viscosity Evaluation

Figure 1B reports the flow sweep curves fitted by the Carreau-Gahleitner model [56], while the values that were obtained by the experimental rheological measurements of η_0_ and η_∞_ are shown in Table 3.

All of the tested solutions showed a low viscosity and typical gel behaviour, with a yield point in the low-shear range; moreover, an evident shear thinning behaviour was observable. Indeed, the samples were characterized by a shear-dependent viscosity (i.e., η decreases quickly with the increasing of the shear rate). F1, F2, and F3 samples showed similar values of η_∞_, but significantly different values of zero-shear rate (η_0_); in particular, in steady state, F3 and F4 appeared to be, respectively, the most and the lowest viscous samples, while F1 and F2 showed intermediate values of η. 

### 2.3. Transmission Electron Microscopy Analysis of the FSs

To evaluate the state of integrity of the collagen fibrils that was extracted with the four different methods, TEM analyses were performed on the four different FSs.

**Figure 2:**
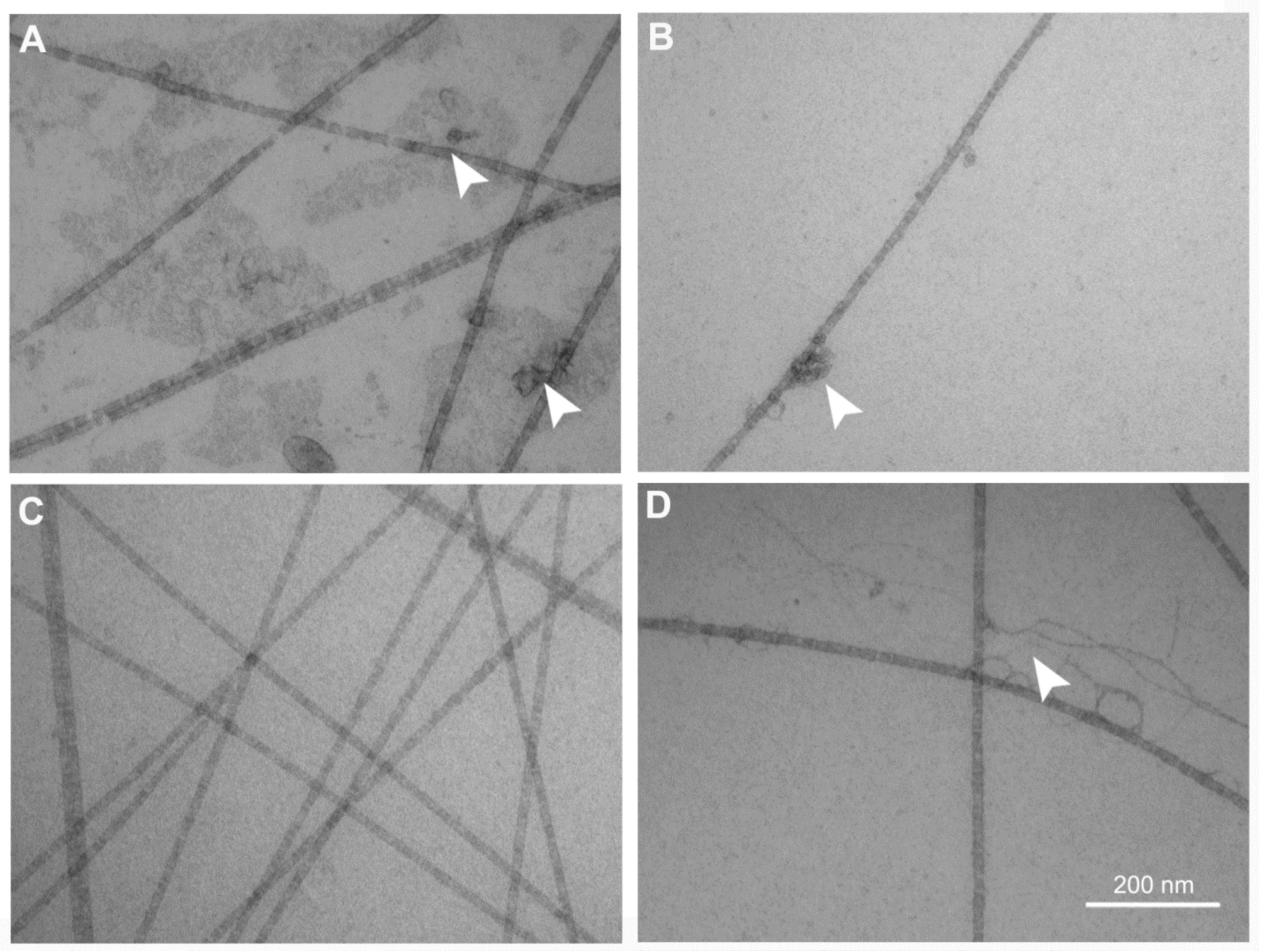
TEM analysis. Representative negatively stained transmission electron microscopy images of the four FSs (F1–F4) taken at 92,000× magnification (scale bar: 200 nm) with a CM10 Philips transmission electron microscope equipped with Megaview 3 camera and Olympus SIS iTEM software for digital image acquisition. (**A**) F1; (**B**) F2; (**C**) F3; and, (**D**) F4. All of the FSs show long unbranched banded fibrils of uniform size and periodicity band pattern. F1, F2, and F4 show small clots and filaments (white arrows) of putative mucopolysaccharides.

At the ultrastructural level, all of the negatively-stained FS samples contained long unbranched single fibrils that was characterized by a periodicity pattern (Figure 2). F1, F2, and F4 samples (panel A, B and D, respectively) showed small clots (white arrows) that were closely apposed to the fibrils and thin curled filaments of *bona fide* mucopolysaccharides (MPS), which were completely absent in the F3 preparation (panel C). Of note, the F2 sample showed many curled fibrils, indicating a possible damage of the structural integrity of the fibrils (not shown). No curled collagen fibrils were observed in the other preparations.

### 2.4. Qualitative Evaluation of the FSs by Histological Methods

**Figure 3:**
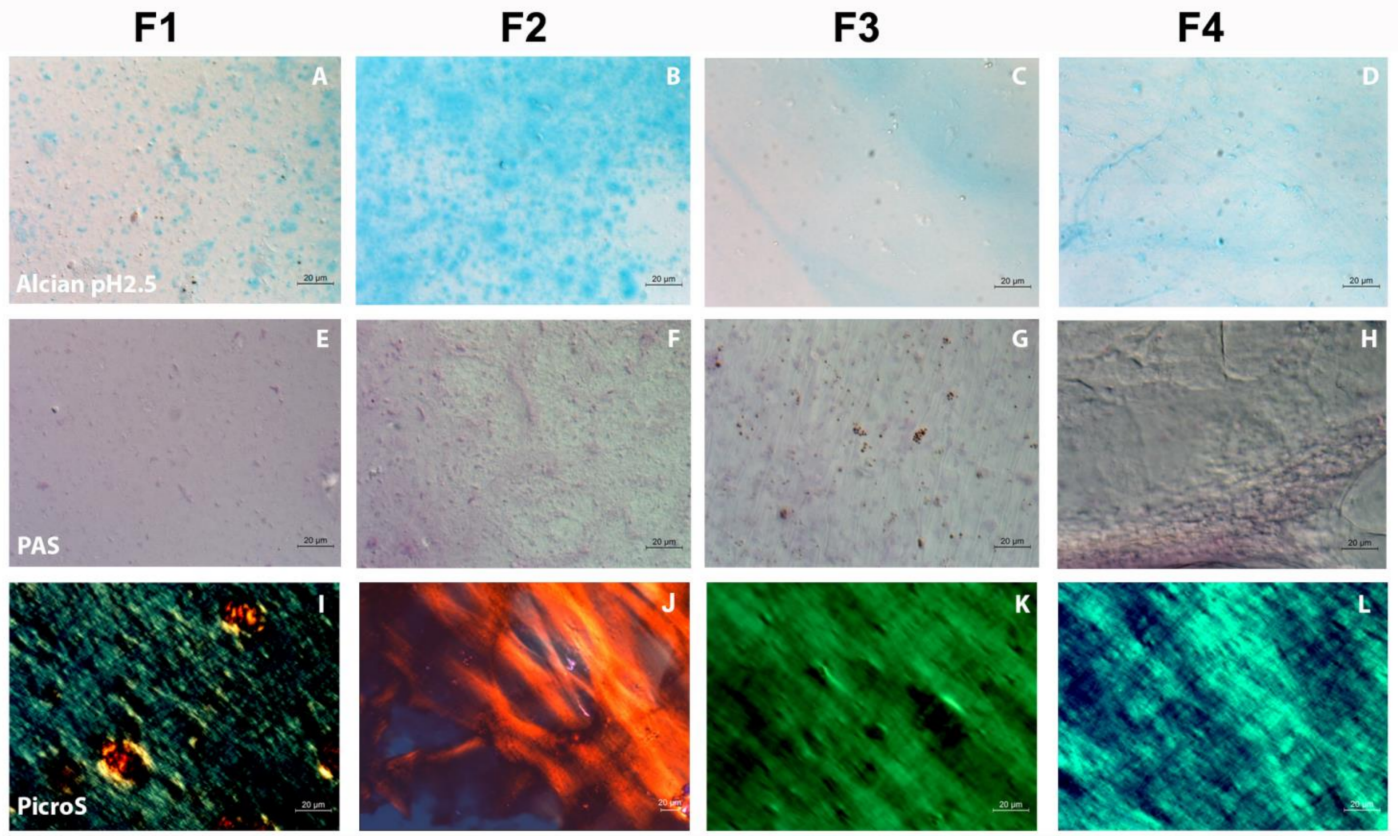
FS histological staining. The four FSs (F1–F4) smeared on histological slides and stained according to four different staining procedures, as described in Section 4.3.4, and observed by optical/polarized microscopy. (**A**–**D**) Alcian pH 2.5 staining, highlighting mucopolysaccharides (MPS) and glycoproteins in blue, by reacting with acidic groups; (**E**–**H**) Periodic Acid Schiff (Hotchkiss–Mc Manus) (PAS)_staining highlighting MPS, glycoproteins, glycolipids, mucins, and polysaccharides such as glycogen in pink/red, by reacting with basic groups; (**I**–**L**) Picro-Sirius Red staining of collagen bundles in different shades of green, red, or yellow, depending on the thickness and the packing of fibres, from thinner to thicker, respectively. Each column of panels represents the various histological stainings of each FS. Scale bars in each panel span 20 micrometer.

FS composition was also qualitatively evaluated by standard histological methods. Different molecular components were highlighted on each FS, smeared and dried on histological slides, and observed in light microscopy (Figure 3). 

Alcian staining (pH 2.5) allowed to highlight in light blue the presence of MPS by reacting with acidic groups. Alcian staining on the smeared FSs showed the following results: in F1 sample (panel A), small and uneven clots of MPS were observable; in the F2 (panel B), a strong coloration of uniformly distributed MPS clots was evidenced; in F3, only a weak MPS staining was detectable in some portions of the sample (panel C); in F4 (panel D), in addition to a weak and diffused coloration, some fibres with an irregular pattern were observable showing the presence of MPS.

PAS staining, on the other hand, highlights in pink/red, the presence of MPS by reacting with basic groups. In the F1 sample (panel E), a poorly concentrated staining of basic little clots was observable. In the F2 (panel F), basic MPS clots were still poorly concentrated. In F3 (panel G), a matrix of transparent/pink fibres was clearly visible by DIC (Differential Interference Contrast), with red clots indicating the presence of basic MPS, likely not being associated with fibres. Finally, in F4 samples (panel H) a weak PAS positive staining of fibrous material was observable, similarly to that observed with Alcian and Picro-Sirius Red (PicroS) in panels D and L, respectively, with the presence of MPS distributed in an irregular pattern.

With the PicroS collagen staining, and through the use of polarized microscopy, it is possible to distinguish small-caliber collagen fibres that result in highlighted in shades of green with respect to thicker fibres that appear to be yellow/red. PicroS staining of the F1 preparation (panel I), showed a discontinuous film of small-caliber fibres. Collagen appeared to be distributed non-uniformly, but in bundles with the presence of small lumps of about 20 µm in diameter showing larger caliber collagen fibres (in red/yellow). Conversely, in the F2 preparation, it was possible to observe an uneven distribution of thicker collagen fibres that are highlighted in red (showed in panel J). Finally, in the F3 and F4 preparations (panels K and L, respectively), the method was able to highlight two continuous fibre films, even if with different chromatic characteristics, pointing out smaller caliber fibres in F3, and thicker in F4.

### 2.5. Sponge Collagen Membrane Production

**Figure 4:**
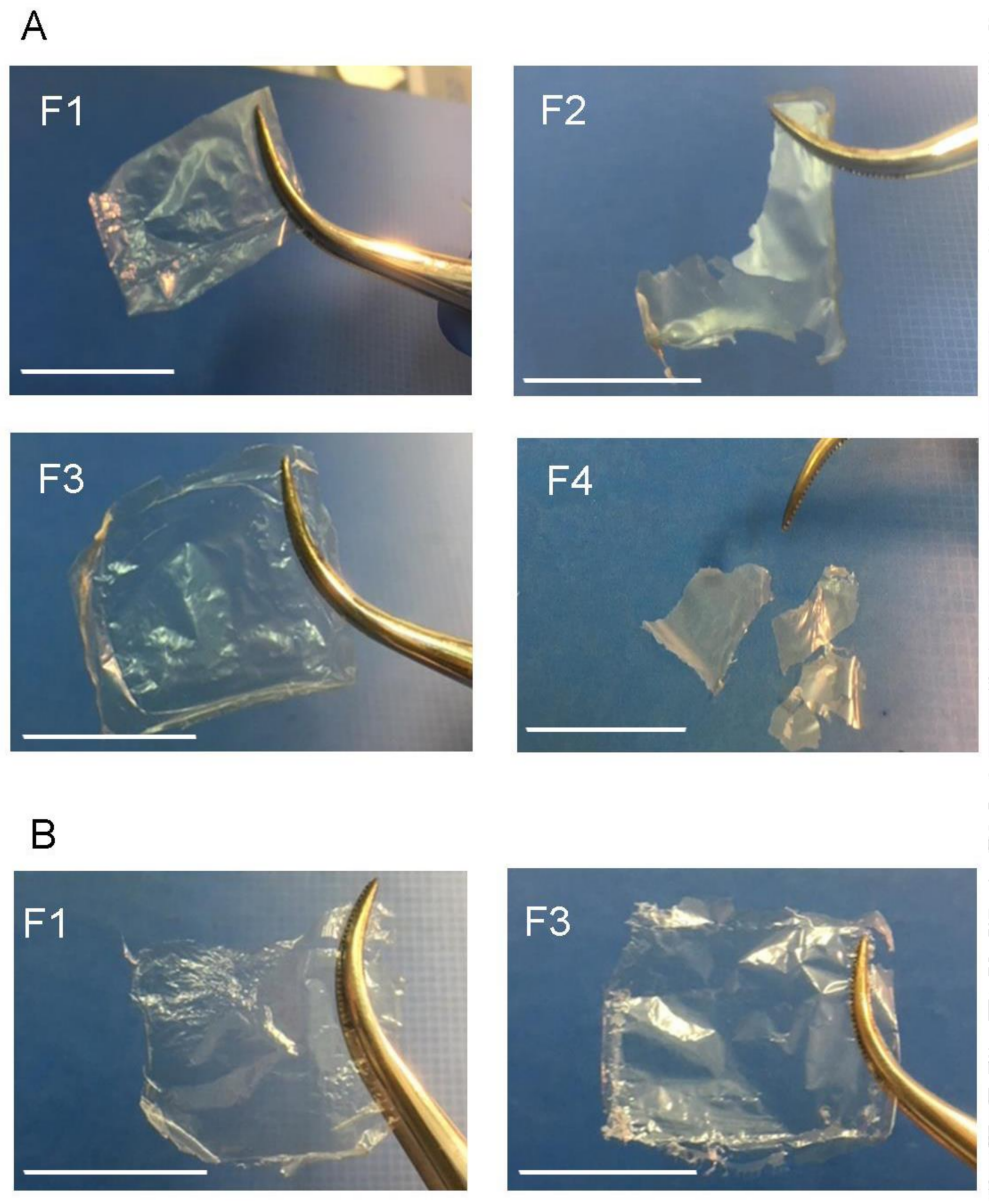
Sponge Collagen Membranes (SCMs). (**A**) SCMs derived from F1–F4 sponge extracts crosslinked with 1-ethyl-3-(3-dimethylaminopropyl) carbodiimide/*N*-hydroxysuccinimide (EDC/NHS) solution; (**B**) not crosslinked SCMs derived from F1 and F3 sponge extracts. Scale bars in each panel span 2.0 cm.

Sponge Collagen Membranes (SCMs) were produced using 2 mg/mL of F1, F2, F3, and F4 fibrillar suspensions in the presence of EDC/NHS crosslinking solution for 4 h at RT. As shown in Figure 4, the only FSs that were able to generate suitable SCMs were F1 and F3 extracts. In fact, F1- and F3-derived SCMs looked like thin, clear, films (Figure 4A). Conversely, using F2 and F4 suspensions, even by increasing the FS concentration (data not shown), it was not possible to obtain any suitable membrane. In fact, when dried the derived films resulted lacking texture, extremely fragile and easy to break. Hence, all of the following characterization analyses were performed only on F1- and F3-derived SCMs named SCM-F1 and SCM-F3, respectively.

As shown in Figure 4B, the membranes were also prepared using not crosslinked F1 and F3 suspensions casting the collagen suspensions without adding the cross-linker. This procedure generated the nc-SCM-F1 and nc-SCM-F3 membranes, respectively, which were macroscopically similar to their crosslinked counterparts.

### 2.6. SCMs Characterization

#### 2.6.1. Mechanical Tests

**Figure 5:**
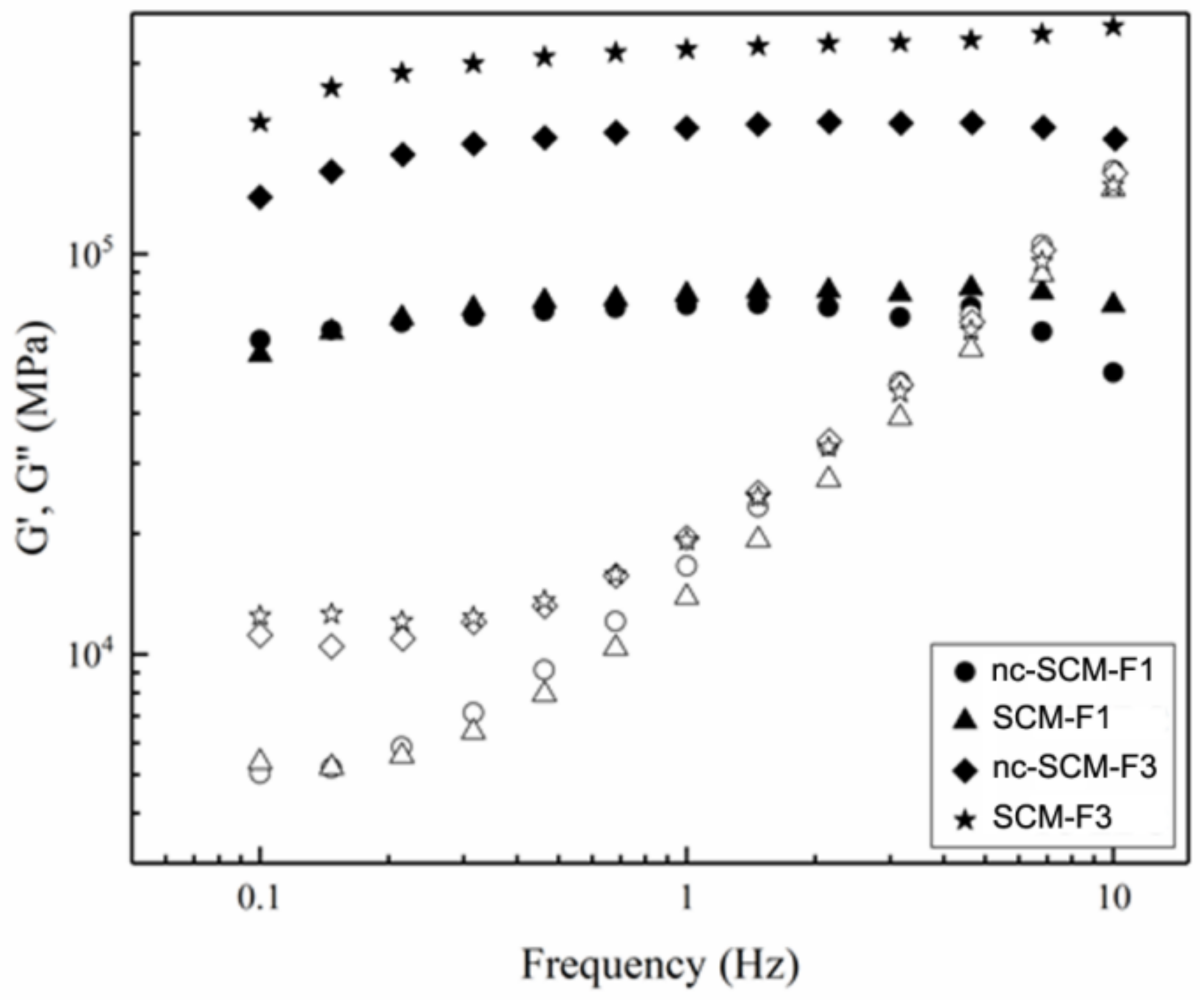
The elastic moduli G′ (filled symbols) and the viscous moduli G′′ (open symbols) of the SCMs (SCM-F1, triangles; SCM-F3, stars) and not crosslinked SCMs (nc-SCM-F1, circles; nc-SCM-F3, diamonds) reported in function of the frequency applied by the rheometer in the dynamic mechanical analysis (for methods see Section 4.5.3).

The elastic moduli G’ (filled symbols) and the viscous moduli G′′ (open symbols) of the F1 and F3 SCMs and nc-SCMs are reported in Figure 5. G′ indicates the capability of the material to store energy and G′′ refers to the capability of the material to dissipate energy. All of the samples that were examined showed relatively high moduli (in the range of 10^4^–10^5^ MPa for the elastic modulus).

As expected, for a gel system, a solid-like behaviour was observable. Indeed, it was possible to note the predominance of G′ upon G′′ at low frequencies in all of the membranes, while G′′ increased on increasing the frequency till reaching a crossover between the moduli. These data indicate that all of the tested samples show an evident elastic behaviour in the frequency range that was studied.

Both SCM-F1 (triangles) and nc-SCM-F1 (circles) showed lower moduli than SCM-F3 (stars) and nc-SCM-F3 (diamonds), indicating, in general, a lower mechanical stiffness. Moreover, as is clearly observable, the crosslinking did not affect the mechanical performance of F1-derived SCMs, with the two samples (nc-SCM-F1 and SCM-F1) showing comparable values of both G′ and G′′. On the contrary, in F3-derived SCMs, a significant difference was observable between the nc-SCM-F3 and the SCM-F3, with an evident increase of the mechanical properties in the crosslinked sample, as evidenced by a higher G′ elastic modulus when compared to the nc-SCM-F3.

#### 2.6.2. In Vitro Resistance to Enzymatic Degradation

**Figure 6:**
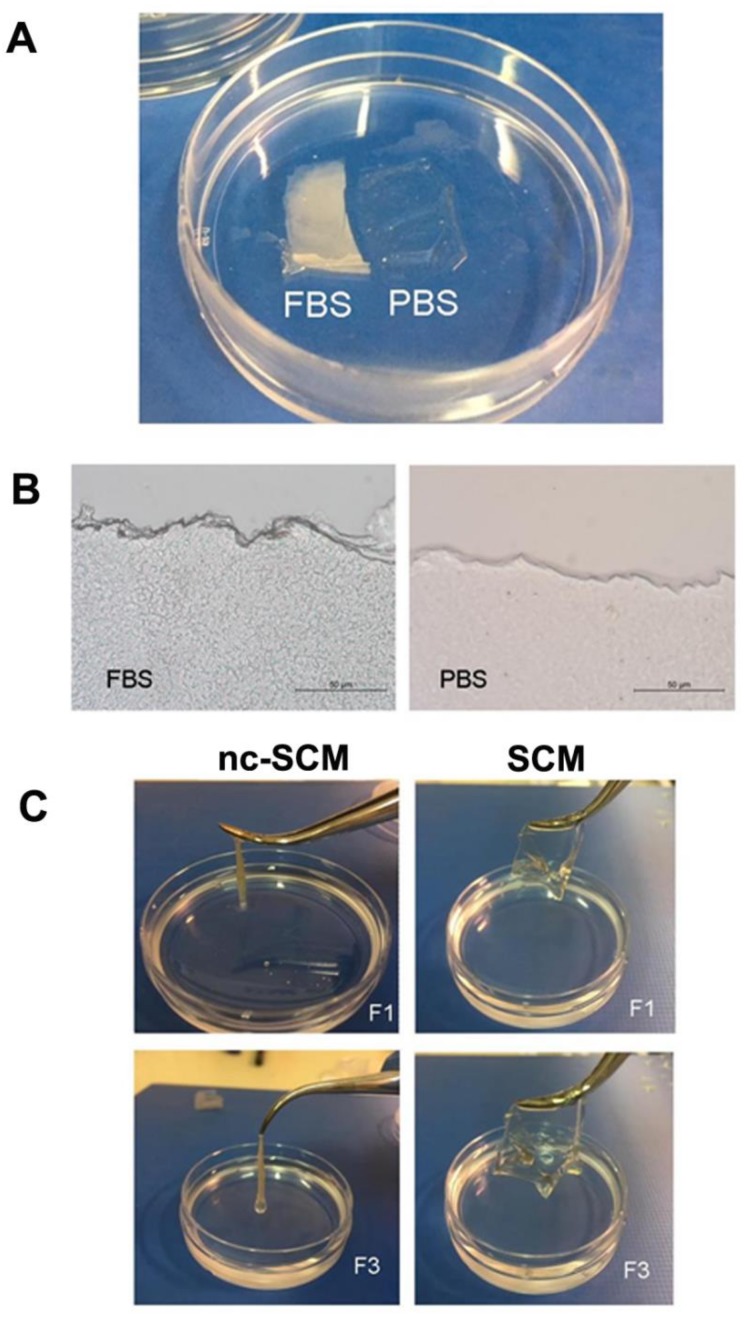
SCM serum interaction and water absorbing capacity. (**A**) SCM-F1 incubated in 1 mL of FBS (left) or PBS (right) for 15 day at 37 °C; (**B**) Stereo-microscope observation of SCM-F1after incubation for 15 days at 37 °C in FBS (left) or PBS (right); scale bar, 50 micrometer; (**C**) Not cross-linked (nc-SCM) and cross-linked SCM derived from F1 and F3 fibrillar extracts after soaking in PBS for 1 h.

In vitro biodegradation of F1 and F3 derived SCMs and nc-SCMs was evaluated by incubation at 37 °C with native fetal bovine serum (FBS) for 15 day and by collagenase digestion for 6 days. The results showed that all of the membranes were completely intact after both of the treatments. This indicates a strong resistance of the marine biomaterials to enzymatic degradation at physiological conditions, even for the not crosslinked versions nc-SCM-F1 and nc-SCM-F3 (not shown). Conversely, a parallel incubation of a commercial mammalian collagen membrane (Bio-Gide^®^, Geistlisch Pharma, Wolhusen, Switzerland), which is usually employed in dental surgery, showed a complete collagenase digestion after 6 days incubation. Furthermore, after FBS treatment, all of the SCMs (crosslinked or not) resulted visibly opaque (Figure 6A) as compared to the negative controls that were incubated in phosphate buffered saline (PBS). Other than opaque, FBS-treated membranes also appeared to be thicker than their controls in PBS, as observed by optical stereomicroscope (Figure 6B). Both features, i.e., opacity and thickness, were likely due to the serum protein adsorption onto the membrane surfaces.

#### 2.6.3. Water Binding Capacity

The water binding capacity (WBC) of SCMs and nc-SCMs was assessed by the evaluation of their weight variation after 1 h soaking in PBS at RT. nc-SCM-F1 and SCM-F1 showed a WBC of 652 ± 35% and of 280 ± 33%, whereas the WBC of nc-SCM-F3 and of SCM-F3 were 701 ± 40% and 420 ± 55%, respectively. Thus, F3-derived membranes showed a slightly higher WBC than F1. Conversely, the commercial not crosslinked collagen membrane Bio-Gide^®^ in the same soaking conditions showed a WBC of 442 ± 41%, which is a value that is significantly lower than the two nc-SCMs, but comparable to the SCMs. 

Once hydrated, it was also clearly observable a significant different behavior of the SCMs with respect to the nc-SCMs. The latter, in fact, although much more hydrophilic than their crosslinked counterparts, completely loosed consistency and resulted in being very difficult to manipulate, as shown in Figure 6C.

#### 2.6.4. Biocompatibility

**Figure 7:**
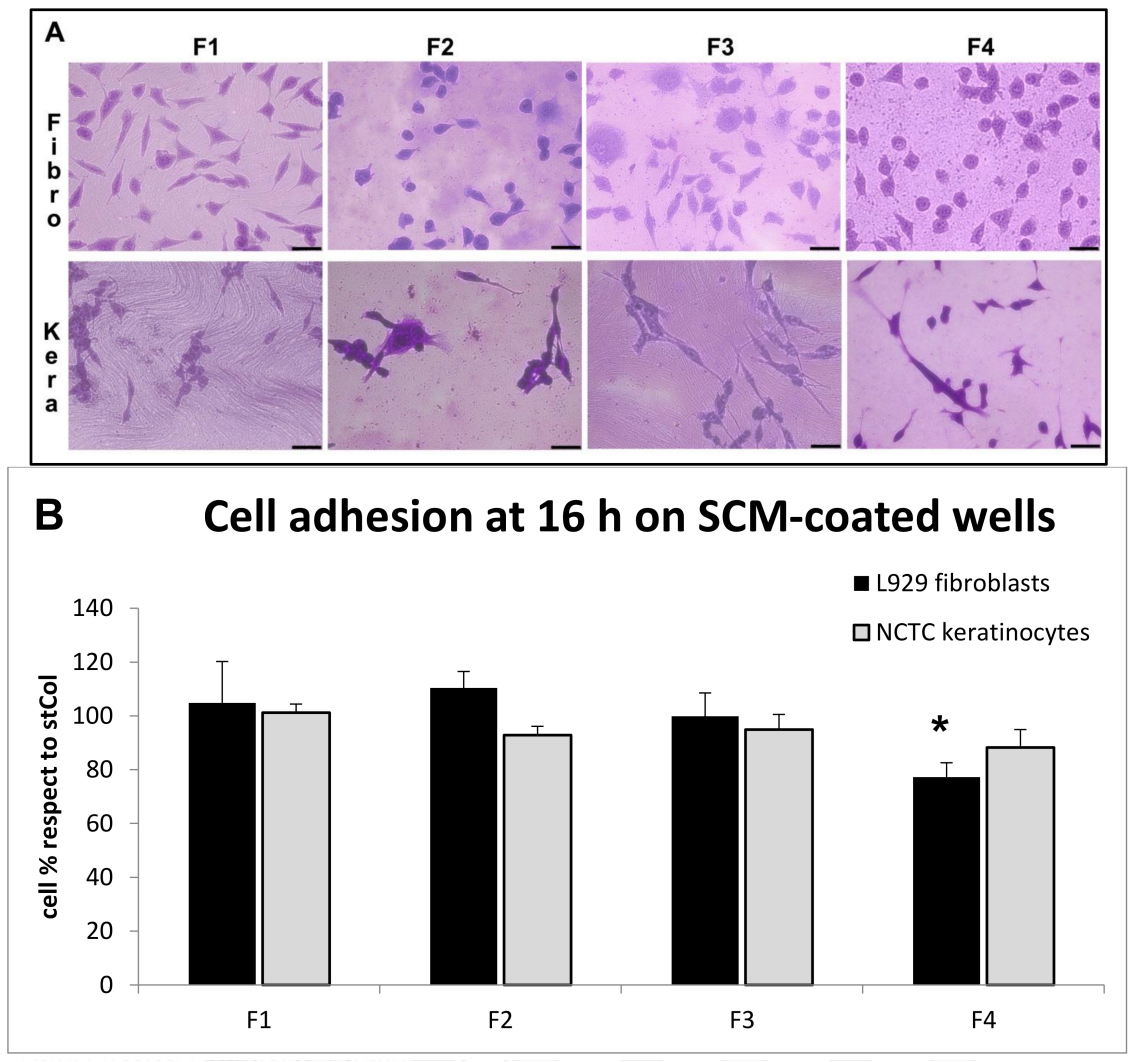
Cell adhesion evaluation. (**A**) Cell adhesion qualitative evaluation, by optical microscopy, of crystal violet stained L929 fibroblasts (first row) and National Collection of Type Cultures (NCTC) keratinocytes (second row) on the four different FS pre-coated plates (F1–F4) after 16 h incubation. Scale bars, 20 micrometer; (**B**) Cell adhesion quantitative evaluation, by MTT test, of L929 fibroblasts (black bars) and NCTC keratinocytes (grey bars) on the four different FS pre-coated plates after 16 h incubation. Results are expressed as cell percentages with respect to controls that were seeded on standard rat tail collagen-coated (stCol) wells and are the mean ± S.D. of two experiments that were performed in quadruplicate. Asterisks indicate significance in Tukey test (black bars ANOVA, *p <* 0.05; Tukey, F4 vs. stCol *p <* 0.05).

To evaluate the biocompatibility of SCM-derived membranes, a fibroblast cell line and a keratinocyte cell line were tested for their ability to adhere and to grow on FS-coated plates using all of the extracts (F1–F4). Cell adhesion was evaluated 16 h after plating both qualitatively and quantitatively by the crystal violet staining and the MTT assay, respectively (Figure 7A,B). The crystal violet qualitative assessment of both fibroblast and keratinocyte cell adhesion revealed that the cell shape, and thus the interaction with the different matrices, was very similar in all of the conditions for both of the cell lines. A slight, qualitative difference could be observed only for the fibroblasts on F4 coating. In particular, a circular shape seemed to be predominant with respect to the physiological spindle/triangular shape typical of these cells, probably indicating a not preferred interaction with this type of coating (Figure 7A). Similarly, the MTT cell viability assay at 16 h of adhesion indicated a reduction of the attached cells only for fibroblasts (Figure 7B, black bars) and only on the F4 coating (21.75% cell reduction as compared to controls). These results confirm a poorer short-term F4 compatibility with respect to the other FSs and to the control, where cells were seeded onto rat tail collagen coated wells, namely stCol.

**Figure 8:**
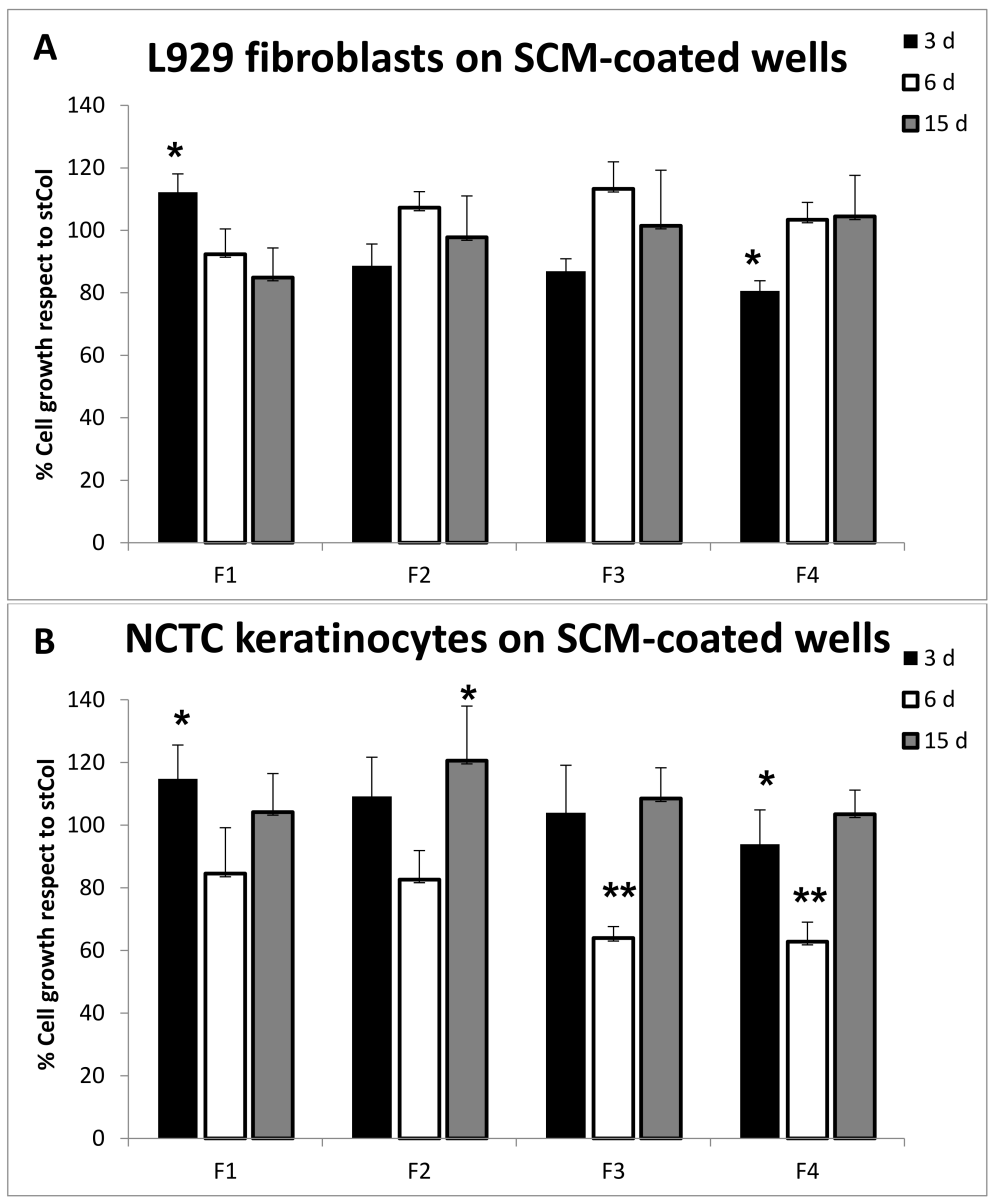
Cell growth evaluation. (**A**) L929 fibroblast cell growth evaluation, by MTT test, on the four different FS pre-coated plates (F1–F4) after 3 day (black bars), 6 day (white bars) and 15 day (grey bars) incubation. Results are expressed as cell percentages respect to controls seeded on standard rat tail collagen-coated (stCol) wells and are the mean ± S.D. of two experiments performed in quadruplicate. Asterisks indicate significance in Tukey test (black bars ANOVA, *p <* 0.0001; white bars ANOVA, *p <* 0.0001; grey bars ANOVA, *p <* 0.05; Tukey vs. stCol: * *p <* 0.05, ** *p <* 0.001, respectively); (**B**) NCTC keratinocytes cell growth evaluation, by MTT test, on the four different FS pre-coated plates after 3 day (black bars), 6 day (white bars), and 15 day (grey bars) incubation. Results are expressed as cell percentages with respect to controls that are seeded on standard rat tail collagen-coated (stCol) wells and are the mean ± S.D. of two experiments that were performed in quadruplicate. Asterisks indicate significance in Tukey test (black bars ANOVA, *p <* 0.00001; Tukey vs. stCol: * *p <* 0.05).

Cell viability and proliferation was also evaluated for longer periods of time on FS-coated plates by using the MTT assay. In particular, both fibroblasts and keratinocytes were evaluated after 3 days, 6 days, and 15 days of cultivation (Figure 8) on the FS-coated plates and compared to control cells that were grown onto rat tail collagen coated wells (stCol). In detail, fibroblasts showed a slight cell number reduction on F4 coating and a slight increase on F1 coating at 3 day with respect to the control (Figure 8A, black bars 19.4% decrease and 12.2% increase, respectively). Conversely, both at 6 day and 15 day of prolonged cell culture (Figure 8A, white bars and grey bars, respectively), no significant differences with respect to the controls grown on rat tail collagen were observed. This indicates a long-term good biocompatibility of the four FS coating for cells of fibroblastic nature. For what concerns keratinocytes, they showed good compatibility and reasonable cell growth, which was comparable to the controls, after 3 day of culture and after prolonged culture for 15 onto all of the FS-coated plates (Figure 8B, black bars and grey bars, respectively). Conversely, a slight, but significant, decrease of cell number at 6 day onto F3 and F4 coatings, as compared to the control, was observed (white bars, 36.0% and 37.2% decrease, respectively), probably indicating that keratinocyte cells undergo a period of adaptation, especially on F3 and F4 coatings, before restarting cell growth.

#### 2.6.5. Environmental Scanning Electron Microscope (ESEM) Analysis 

**Figure 9:**
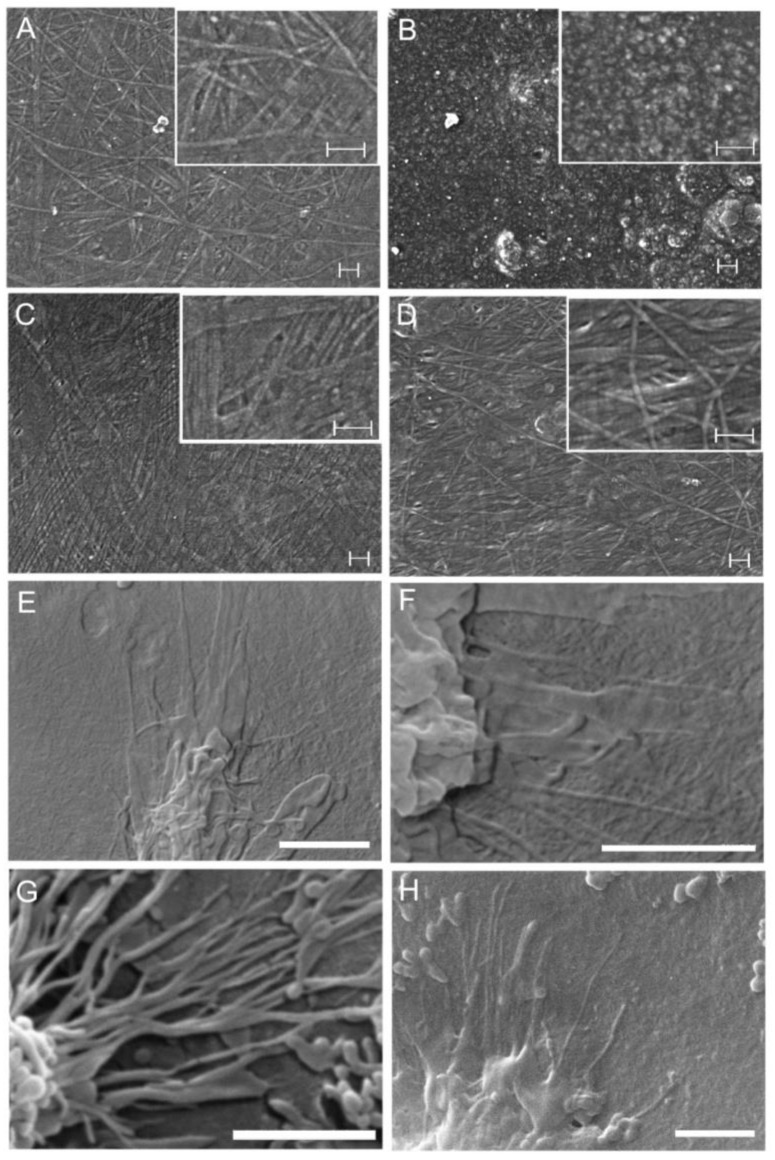
Environmental Scanning Electron Microscope (ESEM) analysis. All crosslinked SCMs (**A**–**D**), dehydrated at critical point and graphite covered, were observed with a FESEM Zeiss SUPRA 40 VP instrument, while only SCM-F1 and SCM-F3, in the presence of fibroblasts and keratinocytes (**E**–**H**) dehydrated at critical point and graphite covered, were observed with an ESEM Vega3–Tescan instrument. (**A**) SCM-F1; (**B**) SCM-F2; (**C**) SCM-F3; (**D**) SCM-F4; (**E**,**F**) Visualization of L929 fibroblasts adhesion to the two SCMs; (**E**) SCM-F1; (**F**) SCM-F3; (**G**,**H**) Visualization of NCTC keratinocytes adhesion to the two SCMs; (**G**) SCM-F1; (**H**) SCM-F3. In (**A**–**D**) scale bars span 200 nm; in (**E**–**H**) scale bars span 2 micrometer.

ESEM analyses were performed on the SCMs that were derived from the four FSs, or fragments thereof for F2 and F4. Furthermore, the analyses were also performed on F1 and F3-derived membranes in the presence of fibroblasts and keratinocytes that were cultured on their sterilized surfaces for 3 day. The ultramicroscopic analysis of the four SCMs alone showed randomly distributed fibril patterns in F1, F3, and F4-derived SCMs (Figure 9A,C,D). Conversely, in F2-derived membranes, the surface that resulted was characterized by an unidentifiable clumped layer (Figure 9B). From F1, F3, and F4-derived SCMs, it was possible to calculate the average of the fibril diameter of *C. reniformis* collagen, which was of 21.08 ± 4.93 nm. Furthermore, in of all the three samples, it was possible to observe either the presence of free single fibrils or bundles of aggregated fibrils forming fibres of higher dimensions. No ultrastructural differences were observed between F1 and F3 crosslinked and not crosslinked membranes (data not shown). In SCM-F1 and F3, the random presence of pores on the surface of the membranes was also observed. SCM-F1 and SCM-F3 pores were measured and they spanned an average value of 6167 ± 2826 nm^2^ and 3211 ± 1494 nm^2^, respectively. The significant difference in the pore dimensions of the two membranes (*p* < 0.005) indicates a higher level of fibre cohesion in F3-derived membranes with respect to F1. 

For what concerns the ultrastructural analysis of cells grown onto the SCM-F1 and SCM-F3 membranes, no significant differences could be observed in the degree of cell adherence and surface contact on both of the SCMs, either for fibroblasts (panel E for SCM-F1 and F for SCM-F3), either for keratinocytes (panel G for SCM-F1 and H for SCM-F3). In fact, all of the images that were obtained by the high resolution cell analysis showed a tight adherence to both SCM surfaces, especially in fibroblasts (E,F), and the presence of several cellular elongated processes taking contact with the SCM surface surrounding the cell body in keratinocytes (G,H). These results suggest that both membranes could have a good biocompatibility.

### 2.7. DPPH Radical Scavenging Activity

Since it is known that the amino acid residues that are present in collagens show a certain level of antioxidant activity [57], the radical scavenging activity of each FS was evaluated using the DPPH standard assay.

**Figure 10:**
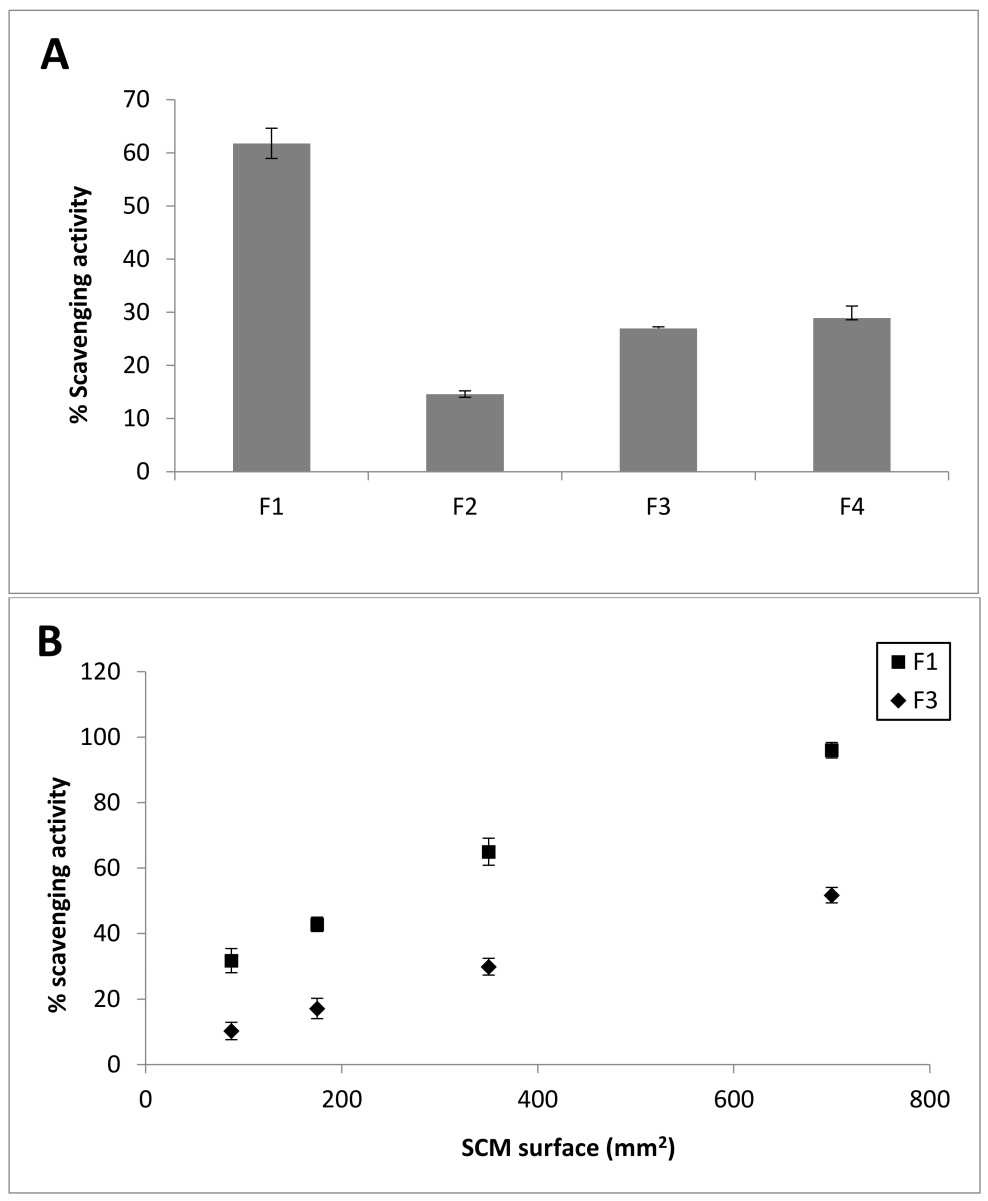
FS and SCM radical scavenging activity. (**A**) Antioxidant activity of the four different FSs (F1–F4) measured by the spectrophotometric DPPH oxidation assay using a concentration of 1 mg/mL of each FS. Results are expressed as percentages of radical scavenging activity based on the inhibition of DPPH oxidation calculated, as described in Section 4.7, and are the mean ± S.D. of two experiments performed in triplicate (ANOVA, *p <* 0.000001); (**B**) Antioxidant activity of SCM-F1 and SCM-F3 measured by the DPPH assay. Results are expressed as percentages of radical scavenging activity in function of the SCM surface area of the two membranes, and are the mean ± S.D. of two experiments performed in triplicate (ANOVA, *p <* 0.000001).

As indicated in Figure 10A, F1 showed the highest radical scavenging activity of 61.78 ± 2.84%, F2 of 14.61 ± 0.61%, whereas for F3 resulted of 26.97 ± 0.23%, and finally F4 showed a radical scavenging activity of 28.89 ± 2.28%. The radical scavenging activity resulted to persist also in F1- and F3-derived membranes (Figure 10B). In this case, the radical scavenging activity was expressed in the function of the membrane surface. In particular, the surface exerting the 50% of the total radical scavenging activity (SA_50_) measured 242.81 mm^2^ for SCM-F1, and 665.84 mm^2^ for SCM-F3.

## 3. Discussion

The ability of the micro-environment and the adhesion substrate to affect various biological processes, i.e., differentiation and proliferation, has been shown in the evolution since the low metazoans [58]. The extracellular matrix (ECM) of multicellular animals is a complex, dynamic system. It is mainly formed by collagen fibrils structured in a highly organized, three-dimensional scaffold supporting cell adhesion and playing a crucial role in differentiation and tissue remodelling [59,60]. Indeed, collagen is considered to be extremely attractive to the manufacturing of biomaterials for TERM applications. The production of collagen-based biomaterials has intensively grown over the past decades. Besides, always new techniques are being developed to improve their performance in terms of mechanical properties and the resistance to enzymatic degradation. Collagenous biomaterials are used as composite materials [61], or as de-cellularized derivatives, obtaining scaffolds that closely mimic the ECM structure and its properties [62]. Marine collagen is considered as one of the most promising sources for these purposes. In particular, the collagen that was derived from the marine sponge *C. reniformis*, due to its dynamic plasticity [43], is particularly interesting for biotechnological applications. In fact, it is considered as a marine biopolymer that is suitable for the production of dynamic biomaterials thanks to its ability to rapidly change stiffness and viscosity similarly to what was observed in the mutable collagenous tissues of echinoderms [63]. Collagenous membranes that are made from ECM extracts, containing native collagen fibrils of echinoderms with good mechanical/biological properties, have been already produced [55]. Conversely, although *C. reniformis* collagen has been studied for a long time [44,45,46], collagenous membranes that are derived from this species have not yet been described. 

The aim of the present work was to obtain marine collagen membranes using intact collagen fibres of this animal. In the production of de-cellularized tissue scaffolds, the purification procedures have a key role, since the removal or maintenance of certain residues may strongly affect their biocompatibility or mechanical properties [64]. In this study, intact sponge collagen fibres were isolated using four different extraction methods (see Scheme 1), and then SCMs were produced from each of them (Figure 4). Finally, the chemical and physical properties of the four extracts were related to the mechanical and biological performances of the respective membranes. These evaluations allowed for selecting the optimal procedures and obtaining the best compromise among yield, 3-D fibre integrity, resistance, and biocompatibility of said membranes.

When comparing the yield values that were obtained in the different FSs (Table 1), the highest total yield and the highest collagen/GAGs recovery was obtained in the F3 suspension. The strongly reducing conditions, due to the presence of 2-mercaptoethanol in the F3 and F4 method, likely contributed to the higher sponge tissue disaggregation and FS yields of these two extraction methods. These results could be explained by *C. reniformis* presence of peculiar short-chain collagen types, which were related to the mammalian type IV, which seem to be involved in the tissue stiffness. In particular, the five conserved cysteines at the C-terminal of these proteins [46], suggest the presence of a disulphide-bridge crosslinking system for a supramolecular arrangement, as already reported in type IV collagen organization in mammalian basal membranes [65]. Thus, the addition of the reducing agent is likely to act at this disulphide-bond level, promoting the complete sponge tissue disruption in the F3 and F4 extraction procedures.

Conversely, for what concerns collagen extraction total yield (Table 1), although the F3 and F4 methods were very similar, the total ionic strength of the extraction buffer seemed to be fundamental to enhancing collagen fibril release. Indeed, the presence of a double NaCl concentration in F3 determined a proportional double release of collagen fibrils from the sponge tissue with respect to the F4 extract.

In the F2 extraction method, where the chaotropic conditions were used in addition to the reducing agent, the total FS yield and low collagen content were significantly different than F3 and F4, which is probably due to the shorter time of incubation in this extraction buffer (i.e., 1 day vs. 4 day, respectively). Conversely, the F1 extraction method provided the highest collagen percentage with respect to the total FS content, indicating trypsin digestion as the best strategy to obtain highly collagen-enriched sponge FSs (Table 2). Furthermore, when comparing the total protein and the collagen percentages in the four FSs (Table 2), we observed that the four methods provided very different values, with F1 suspension showing the highest content of soluble proteins. Conversely, all of the other FSs showed lower and variable percentages of soluble proteins with respect to the total FS. This indicates that these methods (especially F2 and F4) also provided significant amounts of uncharacterized, microscopic materials that are retained in the final FS, despite centrifugation. Notwithstanding the variability of the soluble protein percentage, the collagen/protein ratio seems more homogeneous in the four FSs. Indeed, all of the extraction preparations showed a collagen/protein ratio in a range that was close to 1:2, except for F2, in which the proportion was ≈1:6, indicating that the F2 extraction method retrieves the lowest amount of collagen fibrils from the sponge tissue. Overall, the four methods allowed for obtaining collagen fibrils in good state of integrity, as showed by the ultrastructural qualitative analysis by TEM (Figure 2). Although, in F2 suspension, the presence of many curled fibrils (not shown) likely indicate a slight damage of the extracted collagen. These observations strengthen the idea that the F2 extraction method is less efficient than the others.

The qualitative evaluation of the four FSs by histological staining indicated that the collagen component in F2 preparation had thicker collagen fibres than the other three extracts (Figure 3, PicroS series: panel J vs I-K-L, respectively). Since fibres of such dimensions are supposed to be removed in the centrifugation steps during the extraction, it is probable that the strong PicroS F2 coloration is due to the artificial clotting of the collagen fibres during the acetic acid precipitation step. This again suggests that F2 may not be optimal for subsequent scaffolding procedures. Conversely, a low-diameter fibrillar organization was observable in F1, F3, and F4 PicroS staining, with the bigger collagen bundles that are typically present in *C. reniformis* native tissues [66] being completely removed during the first centrifugation steps.

In *C. reniformis*, tissue collagen fibres are closely associated to complex carbohydrates [44]. Besides, it is known that this component improves the mechanical and the biocompatibility properties of the collagen scaffolds [67]. Thus, the GAG content was also evaluated quantitatively and qualitatively in the four FSs that were obtained from *C. reniformis*. Our data indicate that, even if GAGs are present in low percentages in the four FSs (Table 2), from a qualitative evaluation they seem associated to the collagen fibres extracted with the various methods (Figure 3 Alcian and PAS panels). Although, the F2 suspension differs from the others, showing, respectively, the highest percentage of GAG and the lowest percentage of collagen extracted. In this case, the Alcian histological staining could highlight a significant concentration of GAGs (panel B) probably associated to non-fibrillar proteins. Interesting differences were also observed in the R_C/GAG_, with F1 and F3 showing significantly higher ratios than the other two (Table 2). Considering that these two FSs were the only ones able to generate suitable SCMs and nc-SCMs, we could infer that the ratio between collagen and GAG content in the FS may be determinant for a successful reticulation during the membrane formation. Indeed, our results suggest that a R_C/GAG_ <10 is inappropriate for SCM production.

The viscosity values that were measured in the four extracts (Figure 1B), do not seem to reflect the different collagen or GAG percentages, likely indicating that other factors (i.e., insoluble protein content, electrostatic interactions) could come into play, thus influencing this parameter. These results are in conflict with that observed visually in F1 and F3 suspensions, which appear as dark coloured viscous hydrogels respect to the other FSs. These features probably indicate a higher thixotropic behaviour and the presence of aggregates/micro-gels in solution. Conversely, the hydrogel consistency may be related to the collagen/FS percentage, which is the highest in these two samples, and where the fibrillar network could give a hydrogel aspect to the suspension [68].

SCMs were produced by EDC/NHS crosslinking of the four FSs; however, only F1 and F3 extracts could generate manageable membranes (Figure 4, SCM-F1 and SCM-F3, respectively). Conversely, F2 and F4 tentative membranes lacked structure and texture, were extremely fragile, and were difficult to recover from the silicon mould, also increasing the FS concentration (data not shown). These data indicate that *C. reniformis*-derived FSs with a collagen/FS percentage that is lower than 35% (Table 2) are unsuitable for membrane production. 

ESEM analysis indicated that SCM-F1 and SCM-F3 (as well as also the fragmented SCM-F4) had a fibrillar organization. This further confirms that F1 and F3 extraction procedures were able to maintain an intact fibrillar structure with a significant difference in random pore dimensions between the two (Figure 9 A,C,D). Indeed, the higher mechanical resistance that was showed by SCM-F3 (Figure 5) could be explained by the presence of a smaller mesh of the fibrillar interlace with respect to SCM-F1. 

Using the same FS concentration, F1 and F3 were also casted in silicon moulds without crosslinking. Surprisingly, both F1 and F3 formed collagenous membranes, named nc-SCM-F1 and nc-SCM-F3, respectively. These membranes showed macroscopic (Figure 4B) and ultrastructural (data not shown) features that were similar to their crosslinked counterparts. These data suggest that the collagen extracted from *C. reniformis* is naturally provided with a complex system of preformed fibrillar crosslinks, which may justify either their strong mechanical properties [44] or their insolubility in acidic solutions [49,50].

Another important consideration arising from the results of the membranes mechanical tests is about collagen/total protein content in F1 and F3 suspensions. Due to the higher resistance of SCM-F3 (Figure 5), we can also infer that the higher percentage of non-collagenous proteins present in F3 respect to F1 may have positive impact on the mechanical properties of the sponge FS-derived membranes. Although their exact nature is still unknown, it has been reported that these proteins constitute an amorphous inter-fibrillar matrix in the sponge ECM and are actively involved in the formation of the sponge fibre network [53]. These proteins are susceptible to trypsin treatment, being consequently digested in the F1 extraction procedure, and their absence could help to explain the significant reduction of the mechanical properties that were observed in SCM-F1. Moreover, while no differences were observed for SCM-F1 with respect to nc-SCM-F1 in terms of resistance improvement, a better performance was instead observed in SCM-F3 when compared to nc-SCM-F3. These data indicate that the higher non-collagenous protein content in F3 may participate to the formation of the crosslinks in the membrane, further strengthening this collagenous film. 

Biomaterials originating from collagen still have some limitations to their use in human tissues due to inflammation arising from their biodegradation and relatively short durability [69]. It is known that, both living sponge tissues as well as isolated collagen fibres that are derived from *C. reniformis* are particularly resistant to bacterial collagenases [44]. In our strong experimental conditions (five times higher collagenase concentration than Garrone et al. [44], and 6 day incubation instead of 2 day), all of the membranes, crosslinked or not, maintained their intact structure. This evidence, in addition to confirming previous data on *C. reniformis* collagen, tell us that thin collagen membranes that are derived from this species possess a high stability in strong enzymatic degradation conditions. Conversely, in the same conditions, a commercial not crosslinked collagenous membrane, Bio-Gide^®^, was completely dissolved (not shown). Since this commercial product, which is mainly used in dental surgery, shows an in vivo resorption rate of 2–4 weeks [70], it is reasonable to believe that SMCs may show significantly longer resorption rates. If this was the case, another important requisite for implantable tissue engineering scaffolds would be met by these membranes. Also, extensive membrane treatment (15 day) with native FBS did not show any sign of disaggregation or enzymatic digestion. However, a higher opaqueness and thickness was observed with respect to control membranes that were incubated in PBS (Figure 6A,B). This was likely due to serum protein adsorption on the SCM surface. Indeed, also this feature is considered to be important in the long-term performance of implants [71]. In fact, once the proteins are adsorbed onto the material surface, cell adhesion and growth are facilitated. This adsorbed protein layer can also mediate the type of cells that adhere to the surface, which ultimately can determine the type of tissue that develops. The data collected in the present work indicate that F1 and F3 SCMs, either crosslinked or not, show strong in vitro enzymatic degradation resistance and are able to interact with serum proteins. Thus, they are suitable for the production of biomaterials needing long-term stability in guided tissue and bone regeneration applications.

The ability to bind water is another fundamental aspect of biomaterials. The WBC of SCMs and nc-SCMs indicated a slightly higher WBC in the F3-derived membranes than F1. Moreover, SCMs showed lower WBC than nc-SCMs, likely because most of the functional groups in the membranes are crosslinked and are less available for water interaction. Furthermore, the WBC values of the two nc-SCMs seem quite remarkable if compared to the value of the mammalian collagen not-crosslinked membrane Bio-Gide^®^. The latter, in fact, was 33% and 37% less hydrated than nc-SCM-F1 and nc-SCM-F3, respectively. The increased WBC in the nc-SCMs as compared to the commercial membrane could be explained by the presence, solely in the sponge membranes, of the ECM-derived GAGs. Indeed, their addition has been reported to proportionally improve the membrane’s ability to bind water [72]. However, although nc-SCMs were much more hydrophilic than SCMs and commercial membranes, they appeared to be less easy to manipulate when hydrated (Figure 6C), which is a feature that has to be accurately considered when designing new biomaterials. 

Preliminary biocompatibility assays showed good and encouraging results for a further employment of these biomaterials for medical purposes. Both fibroblasts and keratinocytes were able to adhere and grow on all coated plates when compared to control samples (Figure 7 and Figure 8). In particular, for fibroblasts, the slight cell decrease and the rounded shape onto F4 coating at 16 h indicated a lower short-term biocompatibility with respect to the other FSs and to the controls that were grown onto standard rat tail collagen-coated plates. Conversely, for keratinocytes, a cell decrease was observed only at the 6 day mid-term for the F3 and F4 coatings, maybe indicating the necessity of a period of adaptation onto these two matrices with respect to the other FSs and to the controls. Anyway, this apparent difficulty of adhesion of fibroblasts at 16 h and the growth of keratinocytes at 6 day was only temporary. In fact, long-term analyses at 15 day showed similar growth rates for the two cell lines onto all of the FSs and all closely comparable to the controls (Figure 8). In addition to this, a good cell-SCM surface interaction was observed by ESEM analysis for both cell types on SCM- F1 and SCM-F3, as evidenced by the presence of a tight adherence and a multitude of cell processes interacting with the collagenous membranes (Figure 9E–H). 

Marine sponges are also important sources of bioactive metabolites [36], including compounds with antioxidant properties [73,74], and it is also known that marine collagen-derived peptides possess radical scavenging activity. All of the four FSs that were extracted from *C. reniformis* showed antioxidant properties (Figure 10). These properties, however, did not reflect their collagen percentage; hence, the antioxidant activity may be determined by other molecular types, which were probably co-extracted during collagen fibril isolation. The significantly higher scavenging value of F1 suspension could be due to the absence of reducing conditions in this extraction method, as compared to the other three, which could have partially inactivated the antioxidant component. Surprisingly, the antioxidant properties were also retained in SCM-F1 and SCM-F3, even if reduced with respect to the radical scavenging values of the respective FS. This suggests that these marine membranes are suitable for wound healing applications, for skin repair after superficial cancer treatments, or for the prevention of skin photo-damage and photo-ageing.

In conclusion, as previously reported [20,21,75,76], collagen derived from marine sponges is an extremely performant biopolymer that is suitable for biomedical applications. Here, for the first time, a thorough analysis and chemical characterization of four different sponge collagenous extracts allowed for generating crosslinked thin collagenous membranes from *C. reniformis* demosponge that is suitable for TERM purposes. The two types of SCMs that were obtained showed good mechanical properties, enzymatic degradation resistance, water binding capacity, and biocompatibility. In addition to this, our results demonstrate that it is possible to adapt the extraction procedures in order to alternatively improve the mechanical properties or the antioxidant performances of the derived biomaterials thanks to the versatility of *C. reniformis*-derived extracts.

## 4. Materials and Methods

Chemicals

All reagents were acquired from SIGMA-ALDRICH (Milan, Italy), unless otherwise stated.

### 4.1. Sponge Sampling

Specimens of *C. reniformis* were collected in the area of the Portofino Promontory (Liguria, Italy) at depths of 10–20 m and were transferred in laboratory in a thermic bag. During transport, the temperature was maintained at 14–15 °C. A short-term stabulation was performed, as described in [27]. In particular, the sponges were stored at 14 °C in 200-L aquaria containing natural sea water that was collected in the same area of the Portofino Promontory with a salinity of 37‰ and was equipped with an aeration system. Finally, the sponge specimens were frozen at −20 °C until further processing.

### 4.2. Fibrillar Collagen Suspension Extracts

*C. reniformis* fibrillar collagen suspensions (FSs) were obtained using four different extraction procedures, obtaining, respectively, F1, F2, F3, and F4 extracts. As indicated in Scheme 1, for each procedure, about 25 g of frozen sponge tissue was thawed, extensively rinsed with cool deionized water, cut in small slices, and then minced in a blender in ice, with five volumes of the respective extraction buffer (step 1).

F1 collagen suspensions were obtained, as described in Gross et al. (1956) [53], with some modifications. Briefly, sponges tissue was minced in step 1 in five volumes of 100 mM ammonium bicarbonate pH 8.5, then 0.1% trypsin was added and the sample incubated overnight at 37 °C on a horizontal shaker. Afterwards, the fluid was removed by filtration with a metallic strainer and the solid material was suspended in three volumes of cool deionized water and incubated at 5 °C for three days in a rotary disk shaker aliquoted in 50 mL-tubes. The dark and viscous suspension was then filtered with a metallic strainer and the remained solid material was subjected at a second round of 3 day of water extraction. The viscous fluid that was obtained from the two rounds of water extraction was pooled and centrifuged at 1200× *g*, 10 min at 4 °C, in order to remove cell debris and sand particles. The supernatant fluid containing the collagen suspension was frozen at −20 °C for long-term storage.

F2 collagen suspensions were obtained using the protocol that was described by Diehl-Seifert et al. [52], with some modifications. Here, the sponge specimens were minced in step 1 in five volumes of 100 mM Tris–HCl buffer, pH 9.5, 10 mM EDTA, 8 M urea, and 100 mM 2-mercaptoethanol. The sample was incubated at room temperature (RT) continuously stirring for 24 h. Afterwards, the viscous extract was centrifuged at 5000× *g*, for 5 min at 4 °C. The pellet was discarded and the collagen precipitated from the supernatant by adding 1/3 of its volume of glacial acetic acid, and finally centrifuged at 20,000× *g* for 30 min at 4 °C. The collagen pellet was washed twice with distilled water until the pH was neutral and was finally suspended in 50 mL of 100 mM Tris–HCl buffer pH 9.0, homogenized for 30 s at 24,000 rpm with a T25 basic ULTRA-TURRAX^®^ (IKA^®^-WERKE, Verke Staufen, Staufen im Breisgau, Germany) and stirred overnight at 4 °C. The collagen suspension that was obtained was frozen at −20 °C for long-term storage.

For F3 collagen suspension extraction, as already reported [46], sponge tissue was minced in step 1 in presence of 5 volumes of 50 mM Tris–HCl buffer pH 7.4, 1 M NaCl, 50 mM EDTA and 100 mM 2-mercaptoethanol. The sample was incubated at 17 °C for 4 days in a rotatory shaker disk aliquoted in 50 mL-tubes. The viscous extract was then centrifuged at 1200× *g*, for 10 min at 4 °C in order to remove cell debris and sand particles and the supernatant was extensively dialyzed against deionized water (ratio about 1:20, two changes per day for 5 days at 4 °C) using a 12 kDa molecular weight cutoff membrane tubing, in order to remove excess of 2-mercaptoethanol. The collagen suspension obtained was frozen at −20 °C for long-term storage. 

F4 collagen suspensions were obtained as described by Di Benedetto et al. [55] with some modifications. Sponge tissue was minced in step 1 in five volumes of disaggregating solution that was composed of 100 mM Tris–HCl buffer pH 8.0, 0.5 M NaCl, 50 mM M EDTA, and 0.1 M 2-mercaptoethanol. The sample was incubated at 17 °C for 4 days in a rotatory shaker disk that was aliquoted in 50 mL-tubes. The viscous extract was then centrifuged at 1200× *g*, for 10 min at 4 °C in order to remove cell debris and sand particles, and the supernatant was extensively dialyzed against deionized water, as described for F3 collagen extraction, and at the end, it was frozen at −20 °C for long-term storage. Finally, in order to obtain the fibrillar concentration of each FS extract, 1 mL of suspension was lyophilized and weighted. All of the procedures were carried out two times in duplicate.

### 4.3. FS Characterization 

#### 4.3.1. BCA Total Protein Quantification

0.2 mL of each FS sample (F1, F2, F3, and F4) were centrifuged at 18,000× *g*, for 5 min at RT, the supernatant was discarded and the insoluble collagenous pellet was solubilized in 0.2 mL of 8 M urea pre-heated at 50 °C. The samples were then centrifuged at 18,000× *g*, for 2 min at RT in order to remove any insoluble residues. Total protein content was assayed in the soluble supernatant with Bicinchoninic Acid Protein Assay kit, following the manufacturer’s instructions. Absorbance of each sample was read at 562 nm using a Beckman spectrophotometer (DU 640, Beckman Coulter SpA, Milan, Italy), in comparison to a skin porcine gelatin standard curve. The procedure was carried out in duplicate.

#### 4.3.2. Collagen Quantification

The total collagen present in each FS was determined by the estimation of the hydroxyproline content using a modified method that was based on the Cloramine T reaction [77]. 

0.2 mL of each FS was hydrolyzed with 2 N NaOH by autoclaving at 120 °C for 20 min. Samples were neutralized by adding one volume of 2N HCl and were diluted fourfold in deionized water. The hydroxyproline concentration evaluation was obtained by adding Cloramine T and Ehrlich’s reagent, as described previously [77]. Absorbance of each sample was read at 550 nm using a Beckman spectrophotometer (DU 640), in comparison to a cis-4-hydroxy-l-proline standard curve, and, finally, the content of hydroxylated proline residue was used to infer collagen content of each FS using the proportion factor of 1 g of hydroxyproline per 10 g of collagen [44]. The procedure was carried out in duplicate.

#### 4.3.3. Alcian Blue GAG Assay

The glycosaminoglycan (GAGs) content was measured in each FS by the Alcian blue GAG assay, as described by [78]. To 20 µL of each FS extract, 20 µL of 0.027 M H_2_SO_4_, 0.375% Triton X-100, and 4 M guanidine-HCl were added, and then GAGs were stained with 0.2 mL of working dye solution containing 0.25% Triton X-100, 0.018M H_2_SO_4_ and 0.005% Alcian blue. All samples were incubated 10 min at RT in a horizontal shaker and then centrifuged at 18,000× *g*, for 10 min at 4 °C. The stained GAG pellet obtained was solubilized with 0.4 mL of 4 M guanidine-HCl and the absorbance of each sample was read at 620 nm using a Beckman spectrophotometer (DU 640), in comparison to shark cartilage chondroitin sulfate standard curve. The procedure was carried out in duplicate.

#### 4.3.4 Transmission Electron Microscopy: Negative Staining

FS samples (F1, F2, F3, and F4) were fixed in 2% PFA in PBS for 20 min at RT and were washed out in PBS. 5 µL drops of fixed FS were placed on formwar-coated grids for 20 min. When the suspension was partially dried, grids were washed by touching them three times to the surface of a drop of distilled water. Grids adsorbed with FS samples were then stained with 2% uranyl acetate in 0.15 M oxalic acid for 5 min and an additional 5 min in a 9:1 mixture of 2% uranyl acetate and metylcellulose 25 ctp. FS samples were imaged with a CM10 Philips transmission electron microscope equipped with Megaview 3 camera and Olympus SIS iTEM software for digital image acquisition. Representative images of the four FS preparations were taken at 92,000× magnification (scale bar: 200 nm).

#### 4.3.5. FS Qualitative Evaluation by Histological Methods

100 µL of each FS were smeared in triplicate on histological slides and were dried for 30 min at 37 °C. The slides were stained similarly to standard histological sections with various methods, (all products by Bio-Optica SpA, Milan, Italy): Periodic Acid Schiff (Hotchkiss-Mc Manus) (PAS) that produces a red staining reacting with glycol-containing cellular elements, e.g., glycogen or neutral mucopolysaccharides; Alcian (pH 2.5), whih stains in blue acidic polysaccharides, such as glycosaminoglycans and some types of mucopolysaccharides, and finally, Picro-Sirius Red (PicroS), which stains specifically collagen fibres: in bright-field microscopy collagen appears red, when examined through crossed polarized light the larger collagen fibres are bright yellow or orange, and the thinner ones, including reticular fibres, are green [79]. The sections were observed through a Leica DMRB light and epifluorescence microscope equipped with DIC (Leica microsystems srl, Milan, Italy). Images were acquired using a Leica CCD camera DFC420C.

#### 4.3.6. Rheological Characterization

The rheological measurements were performed with an Anton Paar Physica MCR 301 Rheometer (Anton Paar, GmbH, Ostfildern, Germany), which was equipped with a 50 mm cone/plate geometry (CP50). The viscosity curves were carried out using a shear rate range between 0.1 and 2500 s^−1^, and each sample was tested twice to check for repeatability. The Rheometer was used with a Peltier heating system for an accurate control of the temperature. All of the measurements were performed at 20 °C.

### 4.4. SCM Production

For biocompatibility tests, all four FS were used to directly coat 24-well and 96-well plates. 300 µL (for 24-well plates) or 50 µL (for 96-well plates) of 2 mg/mL of each FS and of a standard rat tail collagen in the presence of 0.01% TritonX-100 were placed on the plates and were left to dry at 37 °C overnight. The coated plates were then incubated with 300 µL (24-well plates) or 50 µL (96-well plates) of EDC/NHS cross-linker solution: 30 mM (EDC 1-ethyl-3-(3-dimethylaminopropyl) carbodiimide/15 mM NHS N-hydroxysuccinimide in MES (N-morpholinoethanesulfonic acid buffer 100 mM, pH 5.5) for 4 h at RT in the dark. Crosslinked coated wells were then washed twice with 0.1 M Na_2_HPO_4_ for 30 min and twice with deionized H_2_O for 15 min, and then finally dried/sterilized with 70% ethanol solution.

SCMs were produced using silicone molds as rectangular (25 × 28 mm) sheets for in vitro biodegradation, water binding capacity, and ultrastructural analyses that were filled with 3.3 mL of 2 mg/mL of each FS and as rectangular (10 × 45 mm) sheets for mechanical tests, filled with 2.25 mL of 2 mg/mL of each FS. The FSs were left to dry at 37 °C, successively incubated, as previously described with EDC/NHS cross-linker solution for 4 h, washed with Na_2_HPO_4_, and deionized H_2_O and finally dried/sterilized with 70% ethanol solution.

Negative controls, lacking the cross-linker step, were prepared as well using 3.3 mL of 2 mg/mL F1 and F3 fibrillar suspensions casted in the same molds without adding the cross-linker solution.

### 4.5. SCM Characterization

#### 4.5.1. In Vitro Enzymatic Resistance

In vitro enzymatic resistance of the F1- and F3-derived SCMs and nc-SCMs was determined by evaluating their stability in native fetal bovine serum (FBS, Euroclone, Milan, Italy) and in the presence of a commercial bacterial collagenase. For the FBS stability test, F1- and F3-derived SCMs (6 mg) were incubated with 1 mL of FBS at 37 °C in a humidified atmosphere for 15 day. The collagenase stability evaluation was determined, as already described [44]. Briefly, collagenase from *Clostridium histolyticum* in a ratio of 1:10 (enzyme:substrate) was used in 1 mL of PBS at 37 °C for 6 day. The enzymatic solution was refreshed daily to ensure continuous enzymatic activity on the SCMs. As a control, a commercial porcine collagen membrane (25 × 25 mm), called Bio-Gide^®^ (Geistlich Pharma AG, Wolhusen, Switzerland), which is widely used in dental and bone surgery, was submitted to digestion as well. Experiments were performed three times in duplicate.

#### 4.5.2. Water Binding Capacity

The water binding property of SCMs and nc-SCMs, was evaluated according to an already described method [80]. In brief, phosphate buffered saline (PBS, pH 7.4) was used as hydration medium, and the membranes were soaked for 1 h at RT by complete immersion. Then, the surface excess medium was removed by touching to a filter paper and then the SCMs were weighed (wet weight). As a control, the commercial porcine collagen membrane Bio-Gide^®^, with the same surface area of the SCMs and nc-SCMs, was submitted to hydration as well. The water binding capacity (WBC) was determined using the following Equation:

WBC (%) = (Ww − Wd)/Wd × 100.

Experiments were performed three times in duplicate.

#### 4.5.3. Dynamic Mechanical Tests

Dynamic mechanical analysis was performed with an Anton Paar Physica MCR 301 Rheometer (Anton Paar, GmbH, Ostfildern, Germany), using a Solid Rectangular Fixtures (SFR) system. The temperature was set at 20 °C and each sample was tested at least twice. Tests were performed both in Amplitude Sweep and Frequency Sweep modes.

The values of the stress amplitude were checked by means of an amplitude sweep test, with a deformation range (γ) from 0.001 up to 0.1% at a fixed frequency of 0.1 Hz, in order to ensure that all of the measurements were performed within the linear viscoelastic region (LVER). 

In order to obtain information about the storage (or elastic) modulus (G′), the loss (or viscous) modulus (G′′), the complex viscosity (η*) as a function of the frequency and the Frequency Sweep tests in the range 0.01–10 Hz, at a fixed deformation of 0.01% within LVER, were performed. 

The data were collected and analysed using Rheoplus/32 Service V3.40 software.

### 4.6. SCM Biocompatibility Evaluation 

#### 4.6.1. Cell Cultures

The L929 mouse fibroblast cell line and the National Collection of Type Cultures (NCTC) human keratinocyte cell line were obtained from the American Type Culture Collection (LGC Standards srl, Milan, Italy). Cells were cultured at 37 °C in a humidified, 5% CO_2_ atmosphere, in high glucose Dulbecco’s modified Eagle’s medium (D-MEM) with glutamax (Euroclone, Milan, Italy), which was supplemented with 10% FBS (Euroclone) with penicillin/streptomycin as antibiotics.

#### 4.6.2. Cell Growth and Cell Adhesion

To evaluate cell growth on SCM-coated plates, experiments were performed in quadruplicate on 96-well plates. Both L929 and NCTC cell lines were plated at a density of 5000 cells/well on 96-well plates pre-coated with F1, F2, F3, and F4. *C. reniformis* FSs extracts, prepared, as described in Section 4.4. Conversely, controls were grown onto rat tail standard collagen coated plates, prepared, as already described in the same paragraph. Cells were incubated for 3 days, 6 days, and 15 days at 37 °C in complete medium. At the end of the experiments cell viability was assayed by the MTT test (0.5 mg/mL final concentration), as already reported [81]. For the evaluation of cell adhesion on FS-coated wells, experiments were performed in duplicate on 24-well plates. Both L929 and NCTC cell lines were plated at a density of 50,000 cells/well on 24-well plates that were pre-coated with F1, F2, F3, F4 extracts, or rat tail standard collagen as control. Cells were allowed to adhere for 16 h at 37 °C in complete medium and then the MTT test was performed as well to estimate the attached cells when compared to control cells on uncoated wells. Data are means ± S.D. of four independent experiments.

#### 4.6.3. Light and ESEM Microscopy

For image acquisition in light microscopy cells were seeded at a density of 50,000 cells/well on 24-well plates pre-coated or not with F1, F2, F3, and F4 *C. reniformis* FSs, prepared as described in paragraph 4.4, and allowed to adhere for 16 h in complete medium. At the end of the experiment, the cells were washed with PBS to remove floating-unattached cells and stained with crystal violet by standard procedures (0.1% crystal violet in methanol for 30 min, followed by extensive washing with water). For image acquisition, an inverted optical microscope (IX53 Olympus, Tokyo, Japan) was used equipped with a CCD camera (U-LH100HG Olympus, Tokyo, Japan) and the relative software. 

For ESEM observation of mammalian cells adhering to F1 and F3-derirved SCMs, 50,000 L929 fibroblasts or NCTC keratinocytes were seeded or not onto 0.25 cm^2^ ethanol-sterilized membranes and incubated for 3 day at 37 °C in complete medium. At the end of the experiment SCMs were washed with PBS and fixed with with a mixture of 2% paraformaldehyde and 2.5 % glutaraldehyde 7.4 pH for 30 min, washed with PBS, extracted from the well plates, and mounted on a plastic support. 

Specimens of F1, F2, F3, and F4 SCMs alone and of cells adhering to SCM-F1 and F3 were dehydrated by passing through a series of ethanol alcoholic solutions with an increasing concentration of up to 100%. The dehydrated membranes were further dehydrated at critical point, avoiding the use of acetone due to the presence of the plastic support, and then graphite was covered and observed. Observation and acquisition of the images of cells adhering to SCM-F1 and F3 were performed with an ESEM Vega3–Tescan, type LMU (Tescan Brno s.r.o., Brno, Czech Republic) equipped with a microanalyzer system EDS-Apollo_x and EDS Texture And Elemental Analytical Microscopy software (TEAM). Observation and acquisition of the four SCMs per se were performed with a FESEM Zeiss SUPRA 40 VP (Carl Zeiss AG, Oberkochen, Germany) and its associated software. The showed results are representative of three independent experiments.

Physical measurements of the fibrillar diameter and of the pore areas observed in the collagen membranes was performed on the images that were obtained by the FESEM analysis of the various membranes, using the ImageJ free software (Rasband, W.S., ImageJ, U. S. National Institutes of Health, Bethesda, MD, USA, https://imagej.nih.gov/ij/, 1997–2016). Means ± S.D. were calculated on at least 10 measurements of fibril diameter or pore areas performed on each membrane.

### 4.7. DPPH Radical Scavenging Activity

The radical scavenging activity was evaluated on each FS and on SCMs that were obtained from F1 and F3 suspensions. 500 μL of 1 mg/mL of each FS were added to 500 μL of methanol, and then to 250 μL of 0.1 mM DPPH in methanol solution (2,2-diphenyl-1-picrylhydrazyl, Calbiochem^®^, Millipore SpA, Milan, Italy). A negative control sample with deionized water was prepared in the same manner. All of the samples were incubated for 30 min at RT in the dark. Then, the samples were centrifuged at 18,000× *g*, for 3 min at RT, and finally the supernatant was read at 517 nm using a Beckman spectrophotometer (DU 640). In the blank sample, the DPPH solution was substituted with methanol. The antioxidant activity of the samples was evaluated by the inhibition percentage of DPPH radical using the following equation:

DPPH radical scavenging activity (%) = (A0 − A)/A0 × 100%(1)

where A was sample absorbance rate; A0 was the absorbance of the negative control. The procedure was carried out in duplicate.

For the evaluation of the radical scavenging activity of SCM-F1 and SCM-F3, fragments of 87.5, 175, 350, and 700 mm^2^ were immersed in 500 µL of deionized water, and, after 15 min of incubation at RT, the samples were processed, as described above. The DPPH radical scavenging activity values were plotted in function of the SCM surface and the surface value of the 50% of scavenging activity (SA_50_) was consequently calculated.

### 4.8. Statistical Analyses

Statistical analysis was performed using one-way ANOVA plus Tukey’s post-test (GraphPad Software, Inc., San Diego, CA, USA). *p* values < 0.05 were considered to be significant. 

## Figures and Tables

**Table 1:** Yield value for each of the four extraction procedures (F1–F4) of the total FS (column 3), collagen (column 4) and glycosaminoglycan (GAGs) (column 5), expressed as mg/g dry sponge tissue. ^1^ From [53]; ^2^ from [54]; ^3^ from [46]; ^4^ from [55].

Sample #	Extraction Method	mg FS/g Dry Tissue	mg Collagen/g Dry Tissue	mg GAG/g Dry Tissue
F1	0.1% tryp, 2 rounds in H_2_O ^1^	338.27 ± 25.23	192.13 ± 22.15	12.85 ± 5.10
F2	8 M urea, tris 0.1 M, pH 9, βMe, EDTA ^2^	422.83 ± 68.15	24.16 ± 1.30	20.44 ± 8.79
F3	NaCl 1 M, tris 50 mM pH 7.4, EDTA, βMe ^3^	998.35 ± 16.12	355.68 ± 56.01	26.89 ± 7.52
F4	NaCl 0.5 M, tris 0.1 M pH 8, EDTA, βMe ^4^	837.21 ± 75.87	139.02 ± 44.63	19.78 ± 3.67

**Table 2:** Percentage composition of total soluble proteins (column 2), GAGs (column 4) and collagen (column 5) in the four FSs expressed as mg/100 mg of total FS. Percentage composition of collagen (column 3) in the four FSs expressed as mg/100 mg of total soluble proteins. R_C/GAG_ values (column 6) obtained from the ratio between the collagen content and GAG content in the four extracts.

Sample #	% Soluble Proteins/FS	% Collagen/Proteins	% GAG/FS	% Collagen/FS	R_C/GAG_
F1	95.00	57.34	3.80	56.81	14.95
F2	34.00	16.76	4.83	5.73	1.18
F3	64.80	48.08	2.68	35.65	13.23
F4	27.39	60.62	2.36	16.63	6.99

**Table 3:** Rheological measurements values of η_0_ and η_∞_ of the four FSs from which were derived the flow sweep curves shown in Figure 1B.

Sample #	η_0_ (mPa s)	η_∞_ (mPa s)
F1	495	3.84
F2	911	3.93
F3	2412	3.90
F4	336	1.70
